# Metabolic Reprogramming of Macrophages by Biomimetic Melatonin‐Loaded Liposomes Effectively Attenuates Acute Gouty Arthritis in a Mouse Model

**DOI:** 10.1002/advs.202410107

**Published:** 2024-12-24

**Authors:** Chuchu Ma, Yuyu Jiang, Yan Xiang, Chang Li, Xiaoying Xie, Yunkai Zhang, Yang You, Laozhi Xie, Jianing Gong, Yinzhe Sun, Shiqiang Tong, Qingxiang Song, Jun Chen, Wenze Xiao

**Affiliations:** ^1^ Department of Pharmaceutics School of Pharmacy & Shanghai Pudong Hospital Key Laboratory of Smart Drug Delivery Ministry of Education Fudan University Shanghai 201203 China; ^2^ Department of Pathogen Biology Naval Medical University Shanghai 200433 China; ^3^ Naval Medical Center Naval Medical University Shanghai 200433 China; ^4^ Department of Pharmacology and Chemical Biology State Key Laboratory of Oncogenes and Related Genes Shanghai Universities Collaborative Innovation Center for Translational Medicine Shanghai Jiao Tong University School of Medicine Shanghai 200025 China; ^5^ Department of Rheumatology Shanghai Pudong Hospital Fudan University Pudong Medical Center Shanghai 201399 China

**Keywords:** acute gout, biomimetic, macrophage, melatonin, metabolic reprogramming

## Abstract

Gouty arthritis is characterized by an acute inflammatory response triggered by monosodium urate (MSU) crystals deposited in the joints and periarticular tissues. Current treatments bring little effects owing to serious side effects, necessitating the exploration of new and safer therapeutic options. Macrophages play a critical role in the initiation, progression, and resolution of acute gout, with the cellular profiles closely linked to their activation and polarization. This suggests that metabolic regulation can be of significance in managing gouty inflammation. In this study, it is demonstrated that melatonin, a natural hormone, modulates the metabolic remodeling of inflammatory macrophages by shifting their metabolism from glycolysis to oxidative phosphorylation, further altering functions of the pathogenic macrophage. To improve melatonin delivery to the inflamed sites, macrophage membrane‐coated melatonin‐loaded liposomes (MLT‐MLP) are developed. Benefiting from the inflammation‐homing characteristic of macrophage membrane, such engineered liposomes effectively target the inflamed site and demonstrate potent anti‐inflammatory effects, achieving an enhanced amelioration of acute gouty arthritis. In conclusion, this study proposes a novel strategy aimed at metabolic reprogramming of macrophages to attenuate the pathological injuries in acute gout, providing a potential therapeutic strategy of gout‐associated diseases, especially gouty arthritis.

## Graphical abstract

A novel delivery system, MLT‐MLP, utilizes macrophage membrane‐coated melatonin‐loaded liposomes to target gouty inflammation sites. By shifting macrophage metabolism from glycolysis to oxidative phosphorylation, MLT‐MLP alters functions of the pathogenic macrophage, thereby alleviating inflammation. Consequently, MLT‐MLP represents a promising strategy for the treatment of gouty arthritis and provides new insights into the role of metabolic reprogramming in inflammatory diseases.

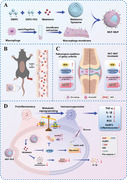

## 1 Introduction

Gouty arthritis is the most common inflammatory arthritis with increasing prevalence and incidence worldwide.^[^ ^1^ ^]^ Gout results from persistent elevation of serum urate levels leading to deposition of monosodium urate (MSU) crystals in joints and periarticular tissues, which triggers recurrent episodes of acute inflammation, known as gout flares.^[^ ^2^ ^]^ The main clinical manifestations of gout are redness, joint swelling, and severe pain, with potential long‐term joint damage and deformity. Currently, the primary approaches to treat gout include uric acid‐lowering therapy (allopurinol, febuxostat, and benzbromarone, etc) and anti‐inflammatory interventions (non‐steroidal anti‐inflammatory drugs (NSAIDs), colchicine, and corticosteroids). However, all of these drugs have serious side effects with long‐term use, including hepatic and renal toxicity.^[^ ^2^ ^]^ Intensive research has focused on understanding the pathogenic processes of gout to improve therapeutic development for better effectiveness and accuracy. Macrophages are considered to be one of the most major cells that initiate and drive inflammation caused by MSU crystals.^[^ ^3^ ^]^ Upon recognizing and phagocytizing MSU crystals by macrophages, the nucleotide‐oligomerization domain‐like receptor (NLR) family pyrin domain‐containing 3 (NLRP3) inflammasome is rapidly activated, leading to the subsequent initiation of the inflammatory protease caspase 1 and the release of interleukin‐1β (IL‐1β), which recruits neutrophils to initiate an inflammatory cascade.^[^ ^4^ ^]^ Based on the acknowledgement, drugs targeting IL‐1β, such as canakinumab, have been developed and complicated in clinical practices. Despite high efficacy against gout flares, their high cost and increasing infection risk have limited their clinical application.^[^ ^5^ ^]^ Hence, there is an urgent need for safer and more effective gout therapeutic drugs.

Currently, metabolic therapy that focuses on regulating the metabolic profiles of immune cells, has attracted increasing attention in multiple of diseases. Instead of broad‐spectrum suppression of immune function, this kind of therapy selectively regulates the immune response, taking the exquisite specificity and intricate regulation of the immune response into account.^[^ ^6^ ^]^ This innovative approach is rooted in the recognition that the activation and differentiation of immune cells are closely linked with metabolic reprogramming — a concept initially described in cancer cells as the Warburg effect, characterized by increased glycolysis and decreased oxidative phosphorylation (OXPHOS).^[^ ^7^ ^]^ In the realm of gout, it is widely acknowledged that metabolic regulation of macrophages profoundly affects the initiation, development, and resolution of gouty inflammation.^[^ ^8^ ^]^ For example, evidence has shown that the metabolic patterns of macrophages are closely related to their diverse phenotype and functions,^[^ ^9^ ^]^ and metabolic regulation is a cornerstone in driving macrophage phenotype switching.^[^ ^10^ ^]^ In general, pro‐inflammatory macrophages (also known as M1 macrophages) utilize glycolysis as their dominant metabolic source, while immunosuppressive macrophages (also known as M2 macrophages) mainly rely on fatty acid oxidation (FAO) and OXPHOS to satisfy their substantial metabolic demands.^[^ ^11^ ^]^ Given that the balance of M1/M2 macrophages is crucial in the progression and resolution of acute gout,^[^ ^12^ ^]^ the therapeutic strategy promoting macrophages from M1 toward the M2 phenotype by regulating the metabolic profiles of macrophages is expected to alleviate the clinical symptoms of acute gout. In addition, recent evidence has shown that NLRP3 inflammation activation is subjected to the dynamic regulation of glucose metabolism and targeting abnormal glycolysis may effectively inhibit NLRP3 inflammation activation.^[^ ^13^ ^]^ Therefore, the treatment to control the metabolic reprogramming of inflammatory macrophages has the potential to prevent and resolute gouty inflammation. Regrettably, few studies targeting metabolic reprogramming have been introduced into the treatment of acute gout as far as we know.

Melatonin (MLT), a natural hormone mainly secreted by the pineal gland, has attracted considerable attention for its multiple pharmacological effects.^[^ ^14^ ^]^ Previous studies have demonstrated that melatonin exhibits powerful antioxidant and anti‐inflammatory effects in a variety of models of acute and chronic inflammatory diseases.^[^ ^15^ ^]^ Regarding gout, research has shown that pinealectomy in mice exacerbates the severity of gouty arthritis, while supplementation with melatonin can partly reverse this effect.^[^ ^16^ ^]^ Moreover, our previous clinical studies revealed that both serum melatonin level and melatonin receptor 2 (MT2) mRNA level in peripheral blood mononuclear cells from acute gout patients were significantly lower than those from healthy individuals, which indicated that melatonin might be involved in the pathogenesis of systemic acute gouty inflammation.^[^ ^17^ ^]^ Hence, melatonin is a promising candidate for acute gout treatment. However, the underlying mechanism remains largely unknown. In this study, we found that MLT could alter the metabolic pattern in the inflammatory macrophages by inducing a metabolic shift from glycolysis to OXPHOS. We further uncovered the previously unknown role of MLT in the metabolic remodeling of inflammatory macrophages during the gouty arthritis.

**Scheme 1:**
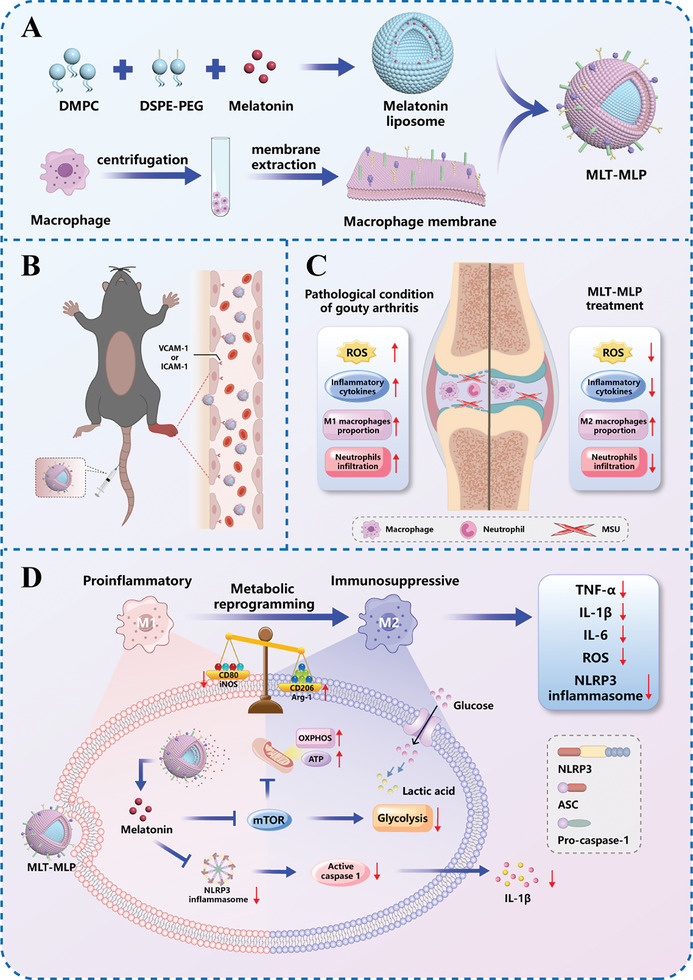
Schematic illustration of the MLT‐MLP delivery system against acute gouty arthritis. A) Preparation of MLT‐MLP. B) Illustration of the targeting mechanism of MLT‐MLP. C) Illustration of pathological condition of gouty arthritis and the therapeutic effect of MLT‐MLP. D) The mechanism of action of MLT‐MLP on macrophages.

Despite the potential in modulating the glycolysis level of macrophages, the clinical application of melatonin has various practical limitations, for example, low water solubility, short half‐life, and lack of specific targeting ability.^[^ ^18^ ^]^ To address these issues, we introduced a biomimetic‐nanoparticle delivery system that consisted of a liposomal core loaded with melatonin and a macrophage cell membrane shell, termed MLT‐MLP (**Scheme** **1**A). Macrophages exhibit an inherent capacity to circumvent clearance by the reticuloendothelial system (RES) and can be persistently recruited through the action of inflammatory chemokines.^[^ ^19^ ^]^ Moreover, benefiting from the effective interaction between the up‐regulated adhesion molecules present on inflammatory vascular endothelial cells^[^ ^20^ ^]^ and integrins/Mac‐1^[^ ^21^ ^]^ on macrophage membrane, MLT‐MLP could achieve inflammation homing during gout flares (Scheme 1B). By loading melatonin, MLT‐MLP exhibited an important role in regulating the metabolic reprogramming of inflammatory macrophages, thereby inhibiting NLRP3 inflammasome activation and facilitating the phenotypic shift of macrophages from the M1 to the M2 phenotype, ultimately resulting in significant therapeutic effects in acute gouty arthritis (Scheme 1C,D). In conclusion, this study not only developed an effective melatonin delivery system for targeted treatment of acute gouty athritis, but also identified a potential pharmacological target for attenuating acute gouty arthritis by controlling the metabolic remodeling of pathogenic macrophages.

## 2 Results

### 2.1 Preparation and Characterization of MLT‐MLP

**Figure 1:**
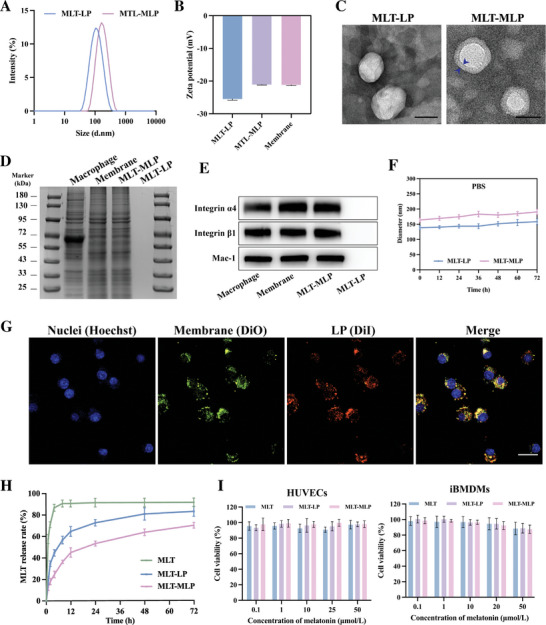
Characterization of MLT‐MLP. A) Size distribution of MLT‐LP and MLT‐MLP. B) Zeta potentials of MLT‐LP, MLT‐MLP, and macrophage membrane (Membrane). C) TEM images showing the morphology of MLT‐LP and MLT‐MLP. Scale bar, 100 nm. D) Protein composition of macrophage, membrane, MLT‐MLP, and MLT‐LP analyzed by Coomassie staining. E) Western blotting analysis showing the representative membrane protein of macrophage, membrane, MLT‐MLP, and MLT‐LP. F) The stability of MLT‐LP and MLT‐MLP in PBS. G) CLSM images of the colocalization of the nuclei (blue), membrane (green), and LP (red). Scale bar, 10 µm. H) Release profiles of MLT, MLT‐LP, and MLT‐MLP in PBS at 37 °C. I) Assessing cytotoxicity in iBMDMs and HUVECs. In all experiments, data are presented as mean ± SD for *n* = 3 biological replicates.

The preparation process of melatonin (MLT)‐loaded macrophage membrane‐coated liposomes (MLT‐MLP) was divided into three steps; (i) preparation of MLT‐loaded liposomes (MLT‐LP); (ii) isolation of macrophage membrane from RAW264.7 cells; (iii) coating MLT‐LP with acquired macrophage membrane. The average hydrodynamic diameter of resultant MLT‐MLP was 163.0 ± 2.0 nm, which was increased by ≈25 nm compared with bare MLT‐LP (138.4 ± 1.1 nm) (**Figure** **1**A; Table S1, Supporting Information). The zeta potential of MLT‐MLP, similar to the charge of macrophage membrane (−21.0 ± 0.21 mV), was −21.2 ± 0.16 mV, which exhibited a slight reduction in negativity in contrast to MLT‐LP (−25.1 ± 0.36 mV) (Figure 1B; Table S1, Supporting Information), indicating the successful coating of the macrophage membrane. The encapsulation efficiency (EE%) and loading capacity (LC%) of MLT‐MLP, determined by HPLC, were 82.91% ± 1.91% and 3.77% ± 0.09%, respectively (Figure S1; Table S1, Supporting Information). To visualize the physical structure of MLT‐MLP, the nanoparticles were examined under transmission electron microscopy (TEM). The imaging revealed a characteristic core−shell structure, with a discernible liposome core and an enveloping layer of macrophage membrane (Figure 1C).

The preservation of protein integrity is essential to the biological functions of MLT‐MLP. Coomassie Brilliant Blue staining showed that most proteins were retained during the process of isolating macrophage membrane. Crucially, the profile of proteins tracked on MLT‐MLP was similar to that of the macrophage membrane, suggesting that the proteins of macrophage membrane could be fully translocated to the surface of MLT‐LP (Figure 1D). Western blot analysis further corroborated the preservation of several key functional proteins specific to macrophages on the surface of MLT‐MLP, including Integrin α4, Integrin β1 and Mac‐1. They are representative proteins for adhesion with endothelial cells (Figure 1E).

The stability of MLT‐MLP was assessed, and the results indicated that MLT‐MLP maintained stability in both PBS and DMEM supplemented with 10% FBS over a 72‐h period (Figure 1F; Figure S2, Supporting Information). Additionally, the colocalization of iBMDMs and MLT‐MLP following cellular internalization was analyzed using confocal laser scanning microscopy (CLSM). Notably, 6 h after iBMDM uptake, significant overlap was observed between DiD (red) labeled liposomes and DiO (green) labeled macrophage membranes, particularly near the Hoechst‐stained nuclei, providing further evidence of the successful coating of the liposomes with the macrophage membrane.

In vitro drug release experiments revealed that MLT‐MLP had better sustained‐release efficiency than free MLT and MLT‐LP, which was likely to be attributed to the coating of the macrophage membrane (Figure 1H). We initially assessed the cytotoxic effects of melatonin on iBMDMs, revealing no significant cellular toxicity until 750 µm (Figure S3, Supporting Information). The cytotoxicity of MLT‐LP and MLT‐MLP on iBMDMs and HUVECs at the concentration from 0.1 to 50 µm was also determined. Encouragingly, the viability of iBMDMs and HUVECs consistently exceeded 80% (Figure 1I), indicating minimal toxicity of MLT‐LP and MLT‐MLP.

### 2.2 In Vitro Targeting Ability of MLT‐MLP

**Figure 2:**
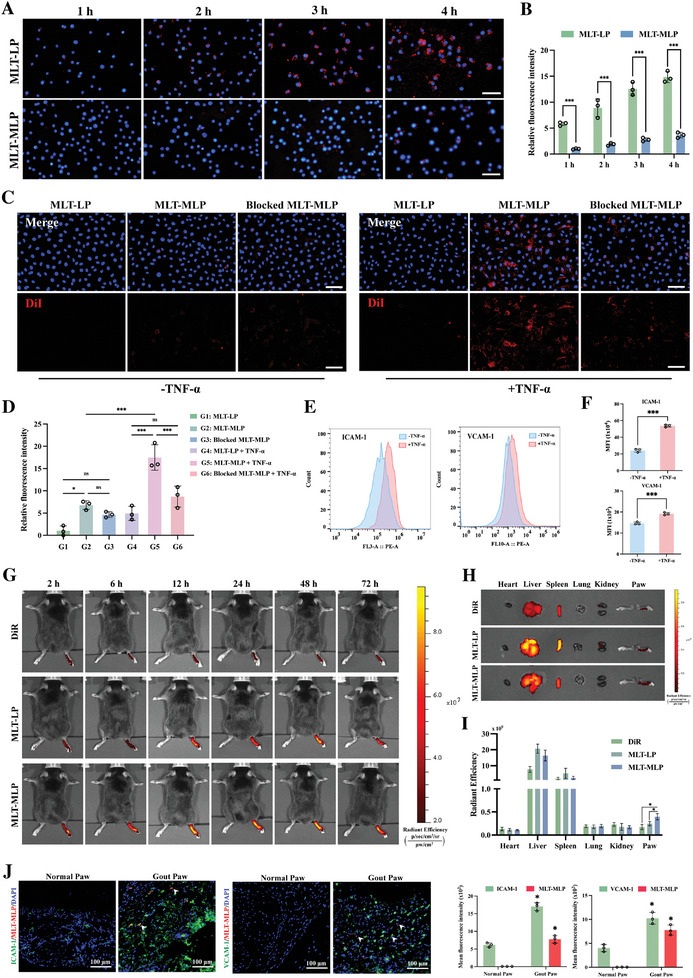
The targeting capability of MLT‐MLP. A) Uptake of DiI‐labeled MLT‐LP and MLT‐MLP (red) by RAW264.7 cells (blue). Scale bar, 100 µm. B) Fluorescence intensity quantitation of nanoparticles (MLT‐LP, MLT‐MLP) corresponding to (A). C) Uptake of different DiI‐labeled nanoparticles by HUVECs with or without TNF‐α stimulation. Scale bar, 100 µm. D) Fluorescence intensity quantitation of different nanoparticles (MLT‐LP, MLT‐MLP and Blocked MLT‐MLP) corresponding to (C). E) Flow cytometry analysis of ICAM‐1 and VCAM‐1 on HUVECs with or without TNF‐α stimulation. F) Quantification of the mean fluorescence intensity of the flow cytometry results corresponding to (E). G) In vivo fluorescence imaging of gout mice after intravenous injection of free DiR, DiR‐labeled MLT‐LP and DiR‐labeled MLT‐MLP. H) Distribution of MLT‐MLP in various organs compared with free DiR and DiR‐labeled MLT‐LP. I) Fluorescence intensity quantitation results corresponding to (H). J) Fluorescence images and intensity of MLT‐MLP (red), ICAM‐1, and VCAM‐1 (green) in normal or gout paws. In all experiments, data are presented as mean ± SD for *n* = 3 biological replicates. ^*^ *p* < 0.05, ^***^ *p* < 0.001; ns, not significant.

Effective escape from clearance by the mononuclear‐macrophage system is conducive to the therapeutic effect of MLT‐MLP. In order to evaluate the immune escape ability of MLT‐MLP, we conducted the phagocytosis assay using RAW264.7 cells.^[^ ^22^ ^]^ DiI‐labeled MLT‐LP and MLT‐MLP were incubated with RAW264.7 cells, and fluorescence images and intensity quantitation results showed that MLT‐LP exhibited significantly higher uptake than MLT‐MLP at 1, 2, 3, and 4 h (**Figure** **2**A,B), indicating that liposomes were endowed with immune escape ability by macrophage membrane, which was beneficial to prolong the circulation of MLT‐MLP in vivo.

Macrophages effectively target gout sites through inflammation‐induced binding followed by extravasation. This binding occurs at the inflamed pannus of blood vessels associated with gout, where higher expression of adhesion molecules such as ICAMs and VCAMs, facilitate macrophage adhesion.^[^ ^20^ ^]^ Western blot results underscored the presence of significant levels of key adhesion molecules, namely integrin α4, integrin β1, and Mac‐1 in macrophage membrane and MLT‐MLP, suggesting that MLT‐MLP could bind to inflamed vessels, akin to macrophages (Figure 1E).

To investigate the binding capability of MLT‐MLP with inflammatory endothelial cells in vitro, HUVECs were stimulated with TNF‐α (50 ng mL^−1^) to induce inflammation. Subsequently, DiI‐labeled MLT‐LP and MLT‐MLP were incubated with the cells. The fluorescence images and intensity quantitation results clearly demonstrated a significantly higher binding affinity of MLT‐MLP with TNF‐α‐treated HUVECs compared to MLT‐LP, emphasizing the notable enhancement in the binding of activated HUVECs by MLT‐MLP (Figure 2C,D). Concurrently, flow cytometry analysis revealed a significant upregulation of ICAM‐1 and VCAM‐1 expression in HUVECs post‐TNF‐α treatment (Figure 2E,F), which indicated that the molecular interactions involving integrins/Mac‐1 and ICAM‐1/VCAM‐1 might contribute to the binding affinity. To further elucidate the binding mechanism, integrin α4 and Mac‐1 on MLT‐MLP were selectively blocked using specific antibodies. As shown in Figure 2C,D, the binding of blocked MLT‐MLP to activated HUVECs was significantly reduced, underscoring that the binding efficacy of MLT‐MLP to inflammatory HUVECs is attributed to these ligands. These findings suggested that MLT‐MLP could effectively target inflammatory vessels in gout mice.

### 2.3 In Vivo Targeting Ability of MLT‐MLP

Given MLT‐MLP's ability to target inflammatory vessels in vitro, its in vivo targeting capability at gout sites was further substantiated using an acute gout model. Each mouse was characterized by one morbid paw and one normal paw. Intravenous administrations of free DiR, DiR‐labeled MLT‐LP, and DiR‐labeled MLT‐MLP were conducted, followed by in vivo imaging. As shown in Figure 2G, the paws of the mice administered with free DiR exhibited relatively low fluorescence signals. In contrast, the gout‐affected sites in mice treated with MLT‐LP demonstrated a significant fluorescence intensity, potentially attributed to passive targeting mechanisms. Notably, the MLT‐MLP group exhibited the most pronounced fluorescence in the gout paw across all observed time points, extending up to 72 h, with no off‐target accumulation observed in the normal paw. The analysis of tissue distribution further highlighted the superior targeting efficacy of MLT‐MLP toward gout‐afflicted paws in comparison to the other formulations (Figure 2H,I), affirming its enhanced specificity and potential for targeted therapeutic delivery.

To corroborate the in vivo binding mechanism of MLT‐MLP, we undertook a comparative analysis of MLT‐MLP localization and the expression levels of ICAM‐1 and VCAM‐1 in both healthy and gout mice. As shown in Figure 2J, a pronounced upregulation of ICAM‐1 and VCAM‐1 were observed in the gout‐afflicted paws in contrast to the normal paw. This increased expression correlated with a notably elevated accumulation of MLT‐MLP, whereas a minimal presence of MLT‐MLP was observed in the normal paw. These results verified that the coating of macrophage membrane enhanced the targeting capacity of MLT‐MLP.

### 2.4 Anti‐Inflammatory Effect of MLT‐MLP In Vitro

**Figure 3:**
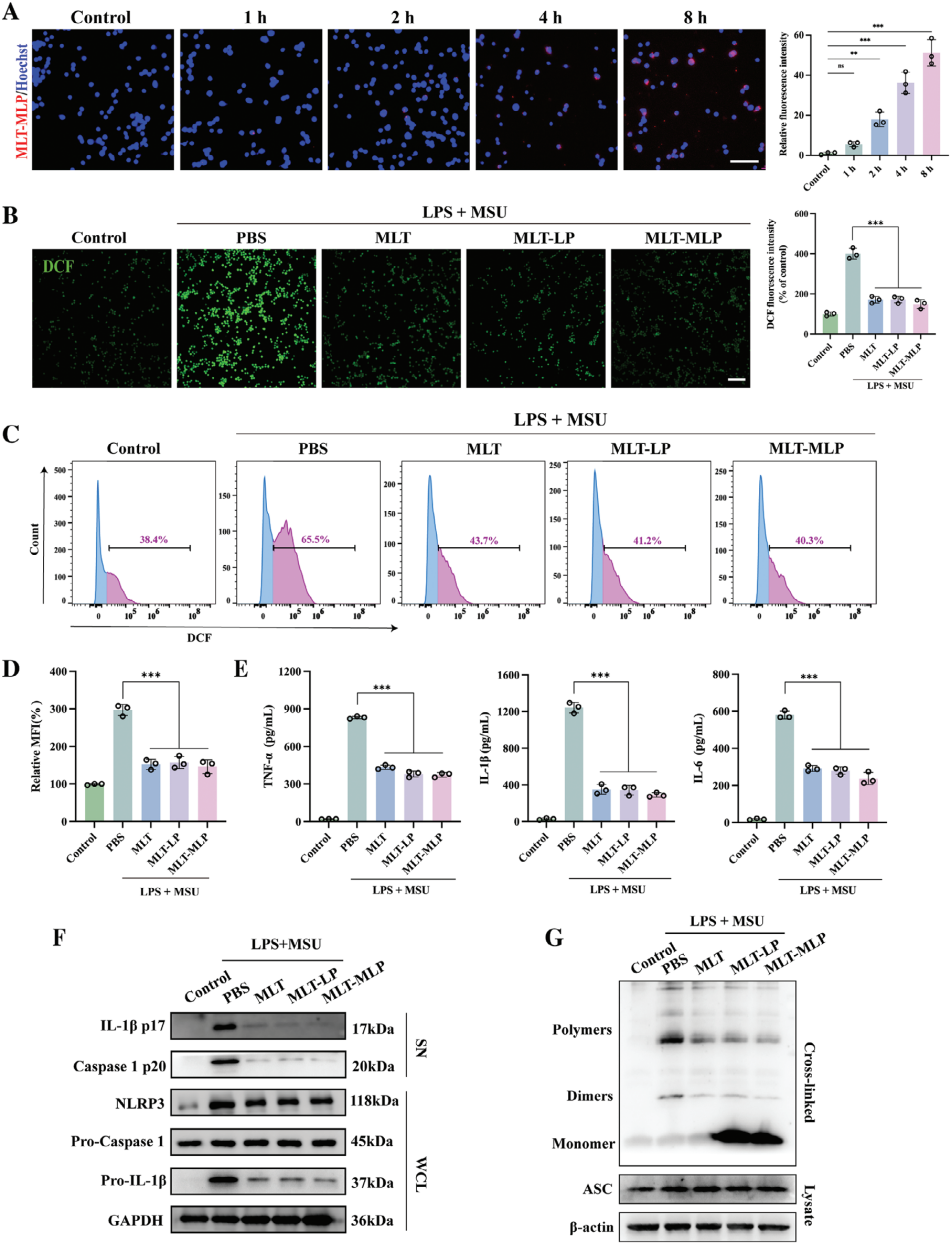
Anti‐inflammatory effect of MLT‐MLP in vitro. A) Uptake of DiI‐labeled MLT‐MLP (red) by iBMDMs (blue). Scale bar, 100 µm. B) Fluorescence imaging of LPS+MSU stimulated iBMDMs stained with the DCFH‐DA probe (green) and quantitative analysis of ROS levels under different treatment conditions. Scale bar, 200 µm. C, D) Quantitative analysis of intracellular ROS levels in iBMDMs using flow cytometry. E) Detection of inflammatory cytokines (TNF‐α, IL‐6, and IL‐1β) produced from BMDMs stimulated with LPS+MSU or not, with different treatments. F) Immunoblot analysis of proteins from the supernatant (SN) and whole cell lysates (WCL) of BMDMs stimulated with LPS+MSU or not, with different treatments.G) Western blot analysis of ASC oligomerization in BMDMs with different treatments. In all experiments, data are presented as mean ± SD for *n* = 3 biological replicates. ^**^ *p* < 0.01, ^***^ *p* < 0.001; ns, not significant.

Given the crucial role of macrophages in the pathogenesis of acute gout, we evaluated the in vitro cellular uptake of DiI‐labeled MLT‐MLP liposomes in inflammatory iBMDMs. As shown in **Figure** **3**A, the fluorescence signal demonstrated a time‐dependent uptake of MLT‐MLP by iBMDMs, which paved the way for MLT‐MLP acting on macrophages.

The deposition of MSU crystals in joints and other tissues triggered the generation of elevated levels of ROS and inflammatory cytokines, further worsening the pathological damage of joint inflammation.^[^ ^23^ ^]^ Moreover, emerging evidence suggests that ROS produced in inflammatory macrophages makes great contributions to NLRP3 inflammasome activation.^[^ ^24^ ^]^ To assess the effect of MLT‐MLP in controlling ROS level, iBMDMs were stimulated with LPS and MSU, along with the treatment of different formulations. Intracellular ROS was detected using DCFH‐DA. Fluorescence microscopy and subsequent intensity quantification revealed an elevation in DCFH‐DA fluorescence within the inflammatory iBMDMs. Notably, treatment with either of MLT, MLT‐LP, or MLT‐MLP resulted in a significant reduction of ROS levels (Figure 3B). Flow cytometry assays achieved similar results (Figure 3C,D), further proving that MLT‐MLP played the important part in inhibiting ROS generation.

To assess the impact of MLT‐MLP on mitigating the activation of inflammatory macrophages, primary mouse bone marrow‐derived macrophages (BMDMs) were established by LPS priming followed by MSU stimulation, with different formulations pre‐treatment. Then the medium of BMDMs under different treatment conditions were collected. As depicted in Figure 3E, the level of inflammatory cytokines, including tumor necrosis factor‐α (TNF‐α), interleukin‐6 (IL‐6), and IL‐1β, were significantly upregulated. All of the anti‐inflammation treatments (MLT, MLT‐LP, and MLT‐MLP) demonstrated a marked decrease in the production of these inflammatory cytokines, indicating their strong anti‐inflammatory potential. The effect of MLT‐MLP on NLRP3 inflammasome pathway was also evaluated. Western blot analysis in Figure 3F revealed that the protein expression of NLRP3 was upregulated upon LPS and MSU stimulation. Additionally, cleavage of caspase 1 (p20), a crucial step in inflammasome activation, facilitating the processing and secretion of IL‐1β, was observed.^[^ ^25^ ^]^ Measurement of the protein expression of caspase 1 (p20) and IL‐1β (p17) in macrophage supernatants showed significant upregulation of both proteins under inflammatory conditions. Further, treatment with either of MLT, MLT‐LP, or MLT‐MLP led to the downregulation of NLRP3 expression, accompanied by noticeable inhibition of caspase 1 cleavage and IL‐1β production, as indicated by the immunoblot assays. Apoptosis‐associated speck‐like protein containing a CARD (ASC) plays a critical role in NLRP3 inflammasome assembly, including the oligomerization of ASC that is essential for inflammasome formation.^[^ ^26^ ^]^ As shown in Figure S4 (Supporting Information), MSU stimulation in LPS‐primed BMDMs induced the formation of ASC speck‐like structures, which were effectively suppressed by treatment with either of MLT, MLT‐LP, or MLT‐MLP. Western blot analysis in Figure 3G further confirmed that MLT‐MLP significantly inhibited ASC oligomerization in inflammatory BMDMs. Collectively, these results indicated that MLT‐MLP could interfere with NLRP3 assembly through inhibiting the ASC oligomerization, which controlled the activation of caspase 1 and release of IL‐1β production, as shown in Figure S5 (Supporting Information). To explore the broader effects of MLT‐MLP on other inflammasome activation, we established in vitro cell models for the absent in melanoma 2 (AIM2) and NLR family CARD domain‐containing protein 4 (NLRC4) inflammasomes. Importantly, MLT‐MLP significantly reduced IL‐1β release in both models, suggesting its capacity to inhibit the activation of AIM2 and NLRC4 inflammasomes. This finding underscores the broad‐spectrum anti‐inflammatory potential of MLT‐MLP (Figures S6 and S7, Supporting Information).

We further evaluated the modulatory effects of MLT‐MLP on the activation of nuclear factor kappa‐B (NF‐κB) signal transduction by immunoblot assays, including the phosphorylation of IKKα/β and P65. In addition, we also evaluated the transcriptional activity of NF‐κB by dual‐luciferase reporter gene assays. As shown in Figure S8 (Supporting Information), Western blot analyses revealed that treatment with MLT‐MLP significantly reduced the levels of phosphorylated IKKα/β and P65 in BMDMs in response to LPS and MSU. Phosphorylation of IKKα/β is a critical step in the NF‐κB signaling pathway, leading to the degradation of IκB and the subsequent translocation of NF‐κB dimers, particularly P65, to the nucleus, where they activate the transcription of pro‐inflammatory cytokines, including TNF‐α.^[^ ^27^ ^]^ The observed decrease in phosphorylated IKKα/β and P65 suggested that MLT‐MLP effectively disrupted this activation of signal cascade. Meanwhile, we also assessed the role of MLT‐MLP in the activation of MAPK pathway, another classical inflamamtory pathway for the indcution of TNF‐α expression, by immunoblot assays. However, no difference was observed in the phosphorylation of ERK and P38, indicating MLT‐MLP had no effects on MAPK pathway. The results from the NF‐κB luciferase activity assays further supported these findings (Figure S9, Supporting Information). MLT‐MLP treatment resulted in a significant reduction in NF‐κB luci activity in the reporter gene assay, which indicated that MLT‐MLP inhibited NF‐κB transcriptional activity in inflammatory macrophages, further confirming its role in inhibiting NF‐κB pathway. Taken together, these results suggested that MLT‐MLP could specifically inhibit NF‐κB pathway, which subsequently led to the significant decrease in TNF‐α production.

In summary, MLT‐MLP effectively reduced ROS production, attenuated the secretion of inflammatory cytokines, and inhibited the assembly of the NLRP3 inflammasome as well as the activation of NF‐κB pathway, thereby demonstrating a potent anti‐inflammatory effect in vitro.

### 2.5 MLT‐MLP Promoted Macrophage Phenotype Shift from M1 to M2

**Figure 4:**
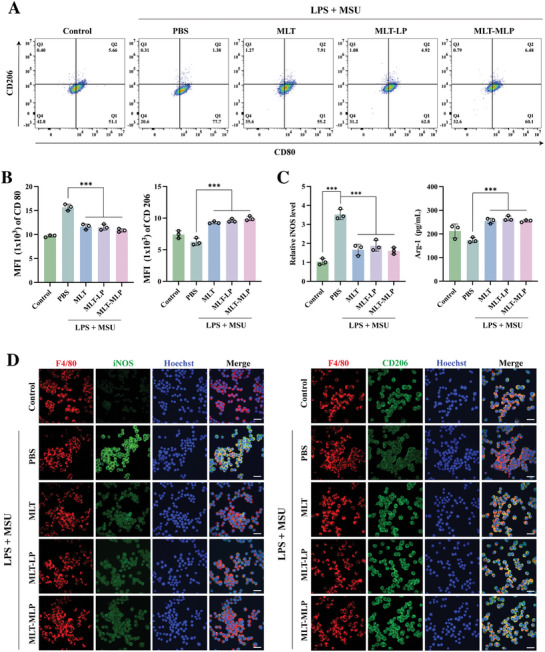
Effect of MLT‐MLP on the polarization of iBMDMs stimulated with LPS+MSU or not, with different treatments.A) Flow cytometry assay of iBMDMs polarization. M1 and M2 phenotypes are distinguished by the presence of CD80 and CD206. B) Quantification of the mean fluorescence intensity of the flow cytometry results corresponding to (A). C) Detection of iNOS and Arg‐1 expressed in iBMDMs with different treatments by ELISA assay. D) Immunofluorescence staining of F4/80 (red, pan‐macrophage marker), iNOS (M1 marker, green) or CD206 (M2 marker, green), and nuclei (blue) on iBMDMs. Scale bar, 50 µm. In all experiments, data are represented mean ± SD for *n* = 3 biological replicates. ^***^ *p* < 0.001.

Emerging evidence underscores a robust association between gout attacks and the imbalance of M1/M2. Repolarization of M1 macrophages into the M2 phenotype has demonstrated treatment efficacy in attenuating acute gout.^[^ ^8a^ ^]^ In order to verify the effect of MLT‐MLP on the phenotype transformation of iBMDMs, M1 markers (CD80) and M2 markers (CD206) on the surface of macrophages were detected by flow cytometry (**Figure** **4**A). Quantitative analysis of fluorescence intensity revealed that the mean fluorescence intensity of CD80 in inflammatory iBMDMs was significantly elevated compared to that observed in the control group, while the expression level of CD206 was moderately reduced. Notably, subsequent treatment with either of MLT, MLT‐LP, or MLT‐MLP markedly reduced the mean fluorescence intensity of CD80 and substantially increased the MFI of CD206. Specifically, in comparison to the model group, MLT‐MLP treatment resulted in a 30.7% decrease in the average fluorescence intensity of CD80 and a 58.7% increase in the average fluorescence intensity of CD206 (Figure 4B). These results demonstrated that MLT‐MLP significantly facilitated the polarization of macrophages from M1 to M2 phenotype.

Inducible nitric oxide synthase (iNOS), indicative of M1 macrophages, and arginase‐1(Arg‐1), a marker highly expressed in M2 macrophages,^[^ ^28^ ^]^ were applied to evaluate the phenotypic shift and showed similar results (Figure 4C). Finally, the results from iNOS and CD206 immunofluorescence staining corroborated the flow cytometry data, illustrating that MLT‐MLP significantly attenuated the fluorescence intensity associated with M1 macrophages while augmenting that of M2 macrophages (Figure 4D). Subsequent semi‐quantitative analysis indicated that, relative to the PBS group, MLT‐MLP treatment led to a 66.4% reduction in iNOS fluorescence intensity and a 62.3% enhancement in CD206 fluorescence intensity (Figure S10, Supporting Information).

In summary, MLT‐MLP demonstrated the capability to repolarize M1 macrophages toward the M2 phenotype, which was beneficial to the relief of gouty inflammation.

### 2.6 Transcriptomic and Proteomic Analyses

**Figure 5:**
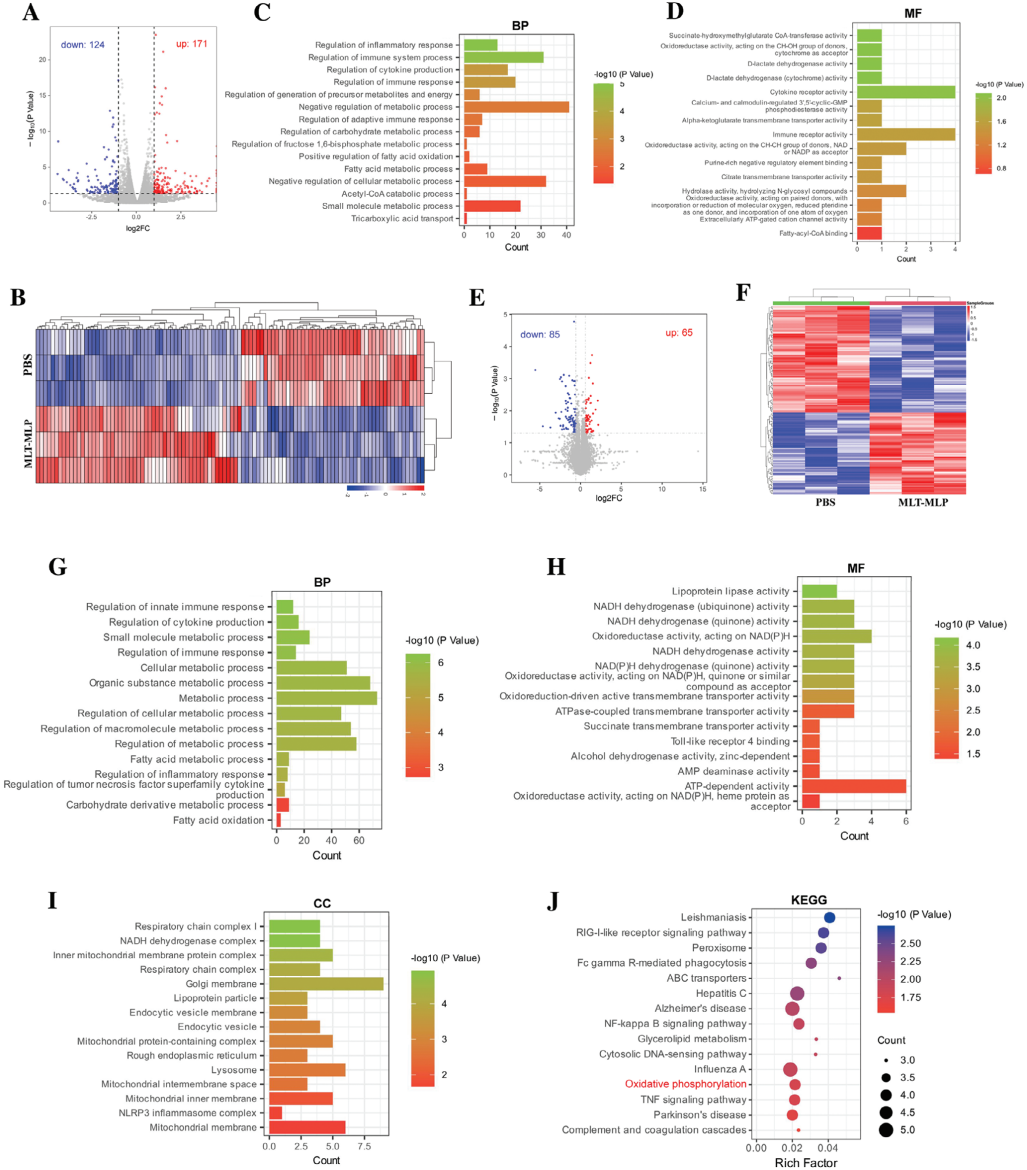
Transcriptomic and proteomic analyses. A) Volcano plots showing DEGs in PBS and MLT‐MLP groups (fold change ≥ 2 and *p* value < 0.05). B) Heatmap analysis of DEGs in PBS group and MLT‐MLP groups. C‐D) GO enrichment analysis of DEGs for BP (C) and MF (D). E) Volcano plots showing DEPs in PBS and MLT‐MLP groups (fold change ≥ 1.5 and *p* value < 0.05). F) Heatmap analysis of DEPs in PBS group and MLT‐MLP groups. G‐I) GO enrichment analysis of DEPs for BP (G), MF (H), and CC (I). J) KEGG enrichment analysis of DEPs.

To further investigate the therapeutic mechanisms of MLT‐MLP, we employed transcriptomics and proteomics techonologies to examine the effects on LPS+MSU‐ stimulated primary mouse BMDMs with MLT‐MLP treatment, using PBS treatment as the control. The transcriptomic analysis indentified 295 differentially expressed genes (DEGs), with 171 genes upregulated and 124 genes downregulated (**Figure** **5**A,B). Gene Ontology (GO) enrichment analyses were subsequently performed. The results of the biological process (BP) analysis indicated that the differentially expressed genes were mainly enriched in “regulation of immune response” and regulation of inflammatory response proveing the previous conclusions that MLT‐MLP had an excellent regulatory effect on inflammatory responses. Additionally, we also observed that the regulated genes were enriched in several metabolism‐related terms, such as “regulation of fructose 1,6‐bisphosphate metabolic process”, “tricarboxylic acid transport”, and “positive regulation of fatty acid oxidation”, which are closely related to carbohydrate metabolism and energy metabolism (Figure 5C). In the molecular function (MF) analysis, the differentially expressed genes played the part in “D‐lactate dehydrogenase activity” and “succinate‐hydroxymethylglutarate CoA‐transferase activity”, both of which are associated with carbohydrate metabolism (Figure 5D). The proteomics analysis revealed 150 differentially expressed proteins (DEPs) by MLP‐MLT treatment, with 65 upregulated and 85 downregulated ones (Figure 5E,F). As shown in Figure 5G, the BP analysis indicated that these proteins were involved in immune regulation, being enriched in terms such as “regulation of innate immune response” and “regulation of cytokine production”. They were also enriched in metabolism‐related terms, including “regulation of metabolic process”, “regulation of cellular metabolic process”, and “fatty acid oxidation”. The MF and cellular component (CC) results showed enrichment in “NADH dehydrogenase activity”, “respiratory chain complex I”, and “respiratory chain complex”, which are associated with mitochondrial oxidative phosphorylation (Figure 5H,I). Furthermore, the Kyoto Encyclopedia of Genes and Genomes (KEGG) pathway analysis of the differential proteins revealed their involvement in “glycerolipid metabolism”, “NF‐kappa B signaling pathway”, and “oxidative phosphorylation” (Figure 5J). These findings indicated that MLT‐MLP not only could play a role in regulating the inflammatory response mediated by macrophages, but also could exhibit the function of modulating cellular metabolism, particular the carbohydrate metabolism and mitochondrial metabolism, thereby influencing the immune activity of macrophages.

### 2.7 MLT‐MLP Regulated the Metabolic Patterns of Macrophages

**Figure 6:**
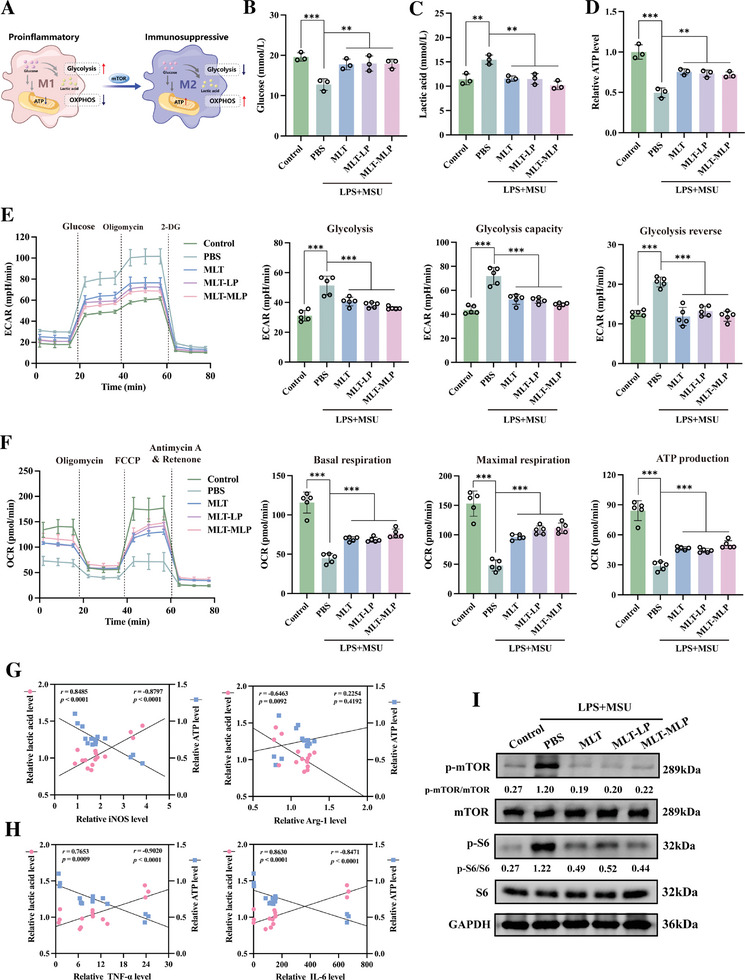
MLT‐MLP regulated the metabolic patterns of inflammatory macrophages stimulated with LPS+MSU through mTOR pathway. A) The scheme of metabolic pattern in M1 and M2 type macrophages. B) Detection of extracellular glucose levels of iBMDMs with different treatments (*n* = 3). C) Detection of extracellular lactic acid levels of iBMDMs with different treatments (*n* = 3). D) Detection of relative ATP production levels of iBMDMs with different treatments (*n* = 3). E) Representative ECAR profiles and corresponding parameter analysis of iBMDMs with different treatments (*n* = 5). F) Representative OCR profiles and corresponding parameter analysis of iBMDMs with different treatments (*n* = 5). G) Correlation analysis of relative lactic acid and ATP levels with iBMDMs polarization parameters levels, including iNOS and Arg‐1. H) Correlation analysis of relative lactic acid and ATP levels with inflammatory cytokines levels, including TNF‐α and IL‐6. I) Representative immunoblots and densitometric analysis for mTOR and p‐mTOR, S6, and p‐S6 in BMDMs stimulated with LPS+MSU or not, with different treatments. In all experiments, data are presented as mean ± SD. ^**^ *p* < 0.01, ^***^ *p* < 0.001.

The metabolic reprogramming of macrophages is crucial in determining their functional phenotypes, impacting their role in immune responses. Studies have shown that macrophages undergo significant metabolic changes in response to stimuli such as lipopolysaccharide. This reprogramming involves shifts in energy utilization pathways, affecting how macrophages mediate immune responses and inflammation.^[^ ^11b^ ^]^ Previous transcriptomic and proteomic analyses showed that MLT‐MLP had the potential of manipulating the metabolic pathways of inflammatory macrophages, so we further performed experiments to explore the alternations of macrophage metabolism by MLT‐MLP. First, we detected the extracellular metabolic substance levels of inflammatory iBMDMs and observed a decrease in extracellular glucose, concurrent with an increase in extracellular lactic acid levels and a decline in ATP levels, indicating an increased glucose consumption and lactic acid generation. Encouragingly, MLT, MLT‐LP, and MLT‐MLP could reverse the abnormal glucose metabolism. Specifically, compared to the PBS group, MLT‐MLP increased the extracellular glucose level, reduced lactic acid production, and increased ATP by 40.3%, 34.2% and 45.1%, respectively (**Figure** **6**B,D).

Then, the Seahorse extracellular flux analyzer was employed to meticulously assess the real‐time alterations in the extracellular acidification rate (ECAR), serving as an index of glycolytic activity, and the oxygen consumption rate (OCR), a measure indicative of OXPHOS. Compared with the control group, the glycolysis rate, glycolysis capacity and glycolysis reserve capacity of inflammatory iBMDMs exhibited a pronounced increase. Conversely, basal respiration, mitochondria‐related ATP production and maximum respiratory rate were significantly decreased, indicating that OXPHOS was inhibited, and glycolysis was dominant. However, compared to the model group, treatment with MLT, MLT‐LP, and MLT‐MLP led to a marked reduction in ECAR parameters, suggesting a decrease in glycolytic activity. In contrast, OCR parameters showed a significant increase, indicating a restoration of OXPHOS (Figure 6E,F). These results revealed that MLT‐MLP had great ability to regulate the metabolic patterns of inflammatory iBMDMs from glycolysis to OXPHOS.

To reveal the correlation between energy metabolism and macrophage polarization, we examined the correlations among lactic acid levels (representing glycolysis), ATP levels (representing oxidative phosphorylation), and the M1 and M2‐specific markers, iNOS and Arg‐1. The results exhibited a robust positive correlation between lactic acid production and iNOS levels (*r* = 0.8485; *p* < 0.0001), coupled with a strongly negative correlation with Arg‐1 levels (*r* = −0.6463, *p* = 0.0092). Furthermore, ATP levels demonstrated a significant negative correlation with iNOS levels (*r* = 0.8797; *p* < 0.0001) (Figure 6G). Delving deeper, we explored the intricate relationship between cellular energy metabolism and the release of inflammatory cytokines. Lactic acid production exhibited a strong positive correlation with the levels of TNF‐α, IL‐6, and IL‐1β (*r* = 0.7653, *p* = 0.0009; *r* = 0.8630, *p* < 0.0001; *r* = 0.7001, *p* = 0.0037), while ATP levels displayed a highly negative correlation with TNF‐α, IL‐6, and IL‐1β levels (*r* = −0.9020, *p* < 0.0001; *r* = −0.8471, *p* < 0.0001; *r* = −0.8349, *p* = 0.0001) (Figure 6H; Figure S11, Supporting Information). These findings suggested that metabolic regulation driven by MLT‐MLP could serve as a booster for altering macrophage phenotype, subsequently influencing the inflammatory cytokines and ROS production.

As a sensor of intracellular energy metabolism, mTOR plays an important role in metabolic regulation and immune response regulation.^[^ ^29^ ^]^ There is evidence showing that activation of mTOR pathway leads to significant metabolic reprogramming from oProductioxidative phosphorylation to glycolysis.^[^ ^30^ ^]^ Western blot analysis showed that mTOR pathway was activated in inflammatory BMDMs, as demonstrated by the increased phosphorylation of mTOR and its downstream effector S6 ribosomal protein (S6). Encouragingly, after either of MLT, MLT‐LP, or MLT‐MLP treatment, the phosphorylation level was down‐regulated (Figure 6I). Overall, these results indicated that MLT‐MLP possibly controlled the glucose metabolism of macrophage through inhibiting the mTOR pathway activation.

### 2.8 Treatment Effects of MLT‐MLP In Vivo

**Figure 7:**
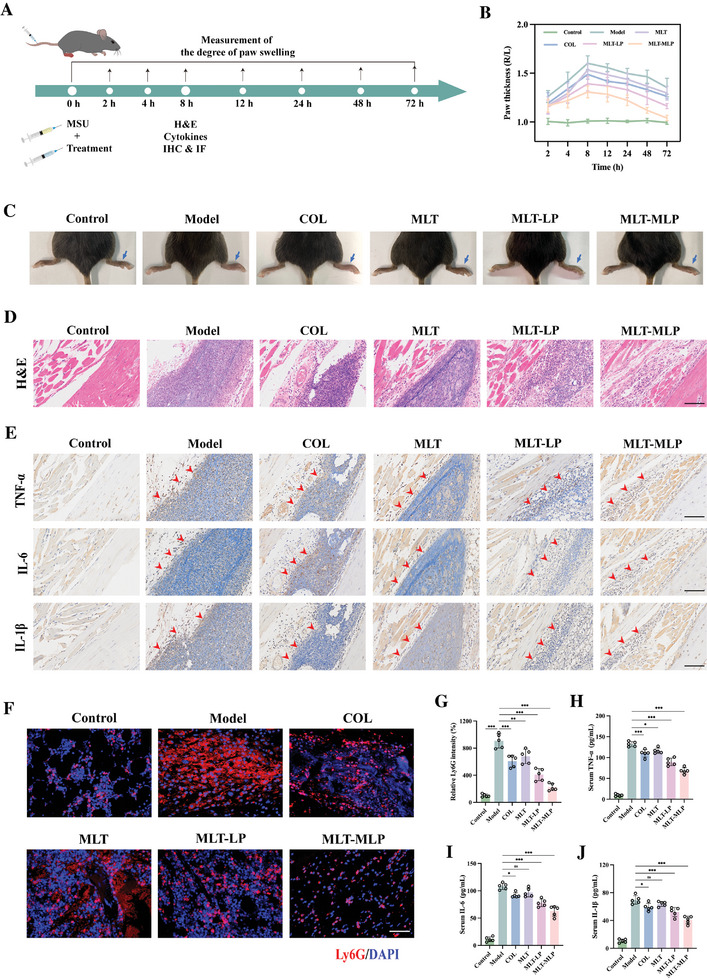
Treatment effect of MLT‐MLP in vivo. A) The experimental scheme. B) Change in paw swelling of gout mice under different treatments. C) Representative photographs of swelling paw obtained at 8 h after MSU injection. D) Photomicrographs stained by H&E. Scale bar, 100 µm. E) Immunohistochemical images of paw tissues in different groups. Scale bar, 100 µm. F,G) Immunofluorescence staining of Ly6G in paw tissues from different groups and corresponding fluorescence intensity quantitation. Scale bar, 100 µm. H–J) Detection of inflammatory cytokines (TNF‐α, IL‐6, and IL‐1β) in the serum of gout mice with different treatments. In all experiments, data are presented as mean ± SD for *n* = 5 biological replicates. ^*^ *p* < 0.05, ^**^ *p* < 0.01, ^***^ *p* < 0.001; ns, not significant.

Based on the above promising results, we conducted an exploration of the therapeutic efficacy of MLT‐MLP in a gouty arthritis mouse model. Following the administration of MSU into the paws, mice were subjected to intravenous injections of saline, free MLT, MLT‐LP, and MLT‐MLP, with the MLT dosage set at 5 mg kg^−1^, respectively. The colchicine was administrated as the same method used in clinic, which is oral administration,^[^ ^31^ ^]^ with a dosage of 0.5 mg kg^−1^. The gout mice treated with intravenous injection of saline were designed as model group. The thickness of paw, serving as a pathological marker for the inflammatory response, was assessed at different time points (**Figure** **7**A). As shown in Figure 7B,C, in the model group, the thickness of the paws exhibited a gradual elevation, peaking at 8 h post‐MSU injection. It also demonstrated that COL, an anti‐inflammatory drug commonly used for gout treatment, and free MLT, could not relieve the acute inflammation of gout significantly. This lack of efficacy is likely due to low bioavailability and poor targeting of free drug, which hindered the accumulation of sufficient drug concentration at the site of gout inflammation. In contrast, MLT‐MLP produced the most favorable therapeutic outcomes, attributed to the inflammation‐homing properties of the macrophage membrane. Histological examination using H&E staining revealed a conspicuous infiltration of immune cells in the paw tissues of the model group. Importantly, MLT‐MLP treatment demonstrated a remarkable reduction in the infiltration of inflammatory cells, affirming its anti‐inflammatory effect (Figure 7D). Immunohistochemical analysis showed a substantial upregulation in the expression of pro‐inflammatory markers, including TNF‐α, IL‐6, and IL‐1β, in the paw tissues of gout mice compared to the control group (Figure 7E). Strikingly, MLT‐MLP treatment significantly downregulated the expression of these inflammatory cytokines, providing further evidence of its ability of controlling inflammation. Gout flare is often accompanied by vasodilatation and rapid recruitment of neutrophils to the site of crystal deposition, which further cause hyperinflammation and aggravate pain and swelling in joints.^[^ ^32^ ^]^ To validate the effect of MLT‐MLP on neutrophil infiltration, immunofluorescence staining of paws associated with gout was conducted using Ly6G antibody (a neutrophil marker). Remarkably, the MLT‐MLP treatment exhibited a significant reduction in the fluorescence intensity of Ly6G, contrasting the elevated signal observed in the model group (Figure 7F,G). Furthermore, the evaluation of serum inflammatory cytokine concentrations, including TNF‐α, IL‐6, and IL‐1β, revealed a marked decrease in the MLT‐MLP group relative to the model group (Figure 7H–J). These comprehensive results not only reaffirmed the pronounced anti‐inflammatory effects of MLT‐MLP but also suggested its systemic impact on circulating inflammatory cytokines, emphasizing its potential as a therapeutic intervention in gout. Taken together, MLT‐MLP showed excellent therapeutic effect in vivo.

### 2.9 In Vivo Biological Safety of MLT‐MLP

**Figure 8:**
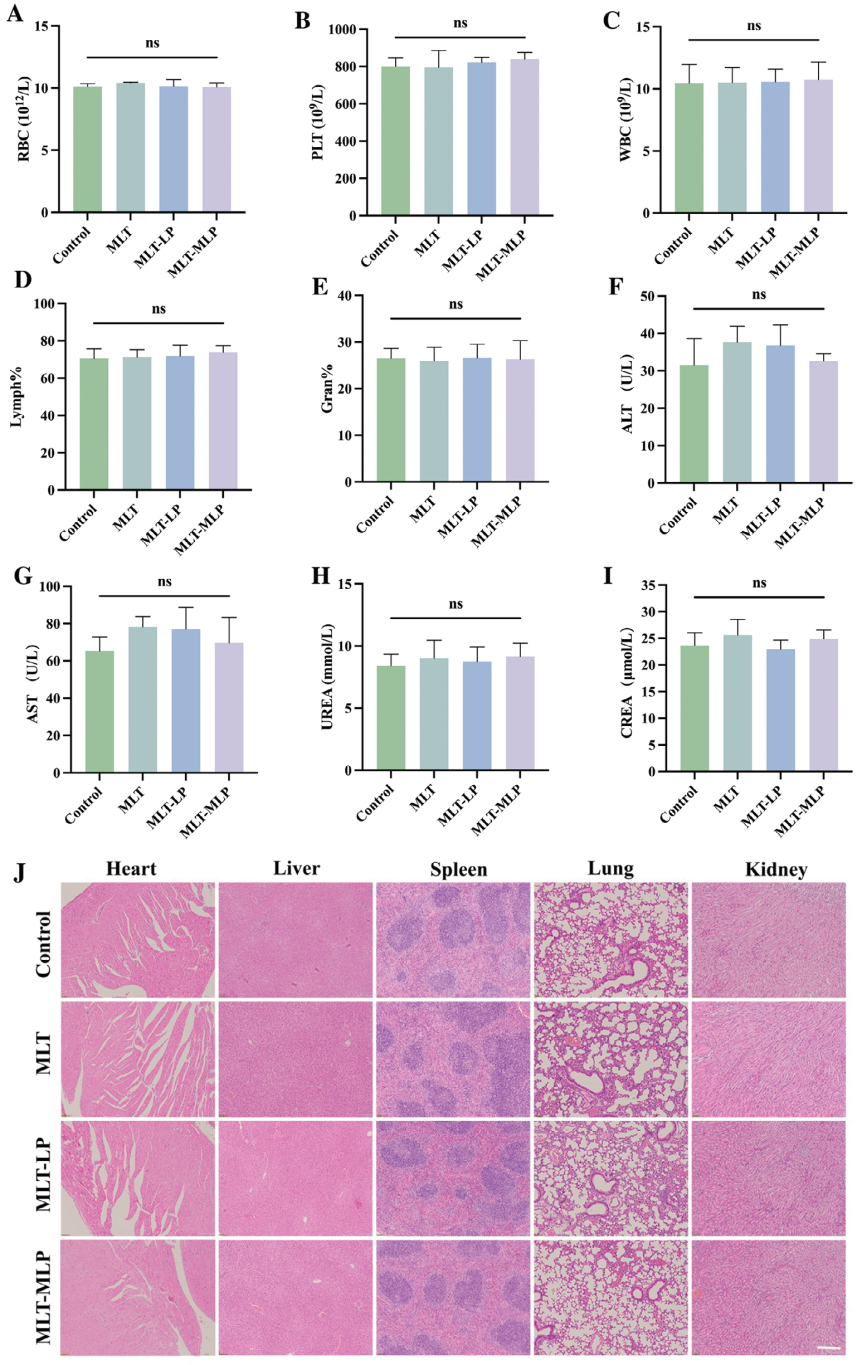
In vivo safety evaluation. A–E) Quantitative analysis of blood routine test, including RBCs, PLTs, WBCs, Lymph%, Gran% (*n* = 5). F–I) The level of AST, ALT, UREA, CREA (*n* = 5). J) Representative images of H&E staining of main organs. Scale bar, 100 µm. In all experiments, data are presented as mean ± SD. ns, not significant.

The investigation into the in vivo safety of MLT‐MLP included comprehensive evaluations through blood routine and serum biochemical assays to assess potential systemic toxicity. Seven days post‐administration when the gouty inflammation almost completely subsides, the red blood cells (RBCs) count (**Figure** **8**A), platelets (PLTs) count (Figure 8B), white blood cells (WBCs) count (Figure 8C), lymphocyte percentage (Lymph%) (Figure 8D), neutrophile granulocyte percentage (Gran%) (Figure 8E) were within normal ranges with no significant inter‐group differences. The level of serum alanine aminotransferase (ALT) (Figure 8F), aspartate aminotransferase (AST) (Figure 8G), urea (UREA) (Figure 8H), and creatinine (CREA) (Figure 8I) revealed no significant differences between the control and treated groups. Moreover, histopathological examination through H&E staining of major organs did not show any discernible signs of organ damage in the treated group as compared to the control (Figure 8J), suggesting that the intravenous administration of the various formulations exhibited good hemocompatibility and biocompatibility in the acute inflammation phase. In the future, more attention should be paied to the long‐term safety concerns, further faciliting the clinial applications.

## 3 Conclusion

Gout is the most common inflammatory arthritis caused by the deposition of MSU crystals in the joints and periarticular tissues.^[^ ^2^, ^4^, ^33^ ^]^ The main anti‐inflammatory medications currently available are NSAIDs, colchicine, and corticosteroids, however, all of these drugs have severe side effects.^[^ ^2^ ^]^ Following the discovery that NLRP3 inflammation activation and IL‐1β release mediate the acute gout initiation, drugs targeting IL‐1β have been developed. Although anti‐IL‐1β treatment has shown significant therapeutic effect, long‐term inhibition of IL‐1β may increase infection risks.^[^ ^5^, ^34^ ^]^ These blunt approaches focus on broad suppression of immune and inflammatory responses, but ignore intricate and specific regulation of the immune responses, potentially disrupting essential homeostatic and protective functions of immune system.^[^ ^35^ ^]^ By contrast, selectively regulating immune processes promises safer and more efficacious outcomes.^[^ ^6^ ^]^

The intimate relationship between the metabolism of immune cells and their differentiation and function underscores the potential of metabolic reprogramming as a strategic approach to modulate immune responses.^[^ ^36^ ^]^ To find such modulator, melatonin, known for its significant antioxidant and anti‐inflammatory attributes,^[^ ^37^ ^]^ was investigated. Our research indicated that melatonin effectively modulated the metabolic landscape of inflammatory macrophages, specifically by inhibiting glycolysis and promoting oxidative phosphorylation. This metabolic adjustment markedly contributed to the relief of gouty inflammation, highlighting melatonin's role as a promising modulator for immune responses. To targeted deliver melatonin to the sites of gout, we introduced a melatonin‐loaded macrophage‐like biomimetic nanodelivery system (MLT‐MLP). Adhesion‐related molecules, including integrin α4, integrin β1 and Mac‐1 were observed on the MLT‐MLP, which would be beneficial to target and bind inflammatory endothelial cells where ICAM‐1 and VCAM‐1 were highly expressed. Subsequently, the enhanced accumulation of MLT‐MLP at gout‐afflicted sites, accompanied by the upregulated expression of ICAM‐1 and VCAM‐1 within the affected region, further corroborated our hypothesis.

The effects of MLT‐MLP on the metabolic profiles of macrophages were further investigated. Attributing to the loading melatonin, MLT‐MLP could significantly reduce glycolysis and promote oxidative phosphorylation. The metabolic pathways utilized by M1 and M2 macrophages underscore their distinct roles in the immune response. M1 macrophages, which are pro‐inflammatory, primarily rely on glycolysis for their energy needs. This metabolic profile supports their role in initiating and sustaining inflammatory responses, including the secretion of pro‐inflammatory cytokines and ROS. Conversely, M2 macrophages, known for their immunosuppressive and tissue repair functions, predominantly utilize OXPHOS for their energy requirements.^[^ ^38^ ^]^ This shift from glycolysis to OXPHOS is beneficial for promoting the transition from a pro‐inflammatory to an anti‐inflammatory state, facilitating the resolution of inflammation. Indeed, MLT‐MLP treatment demonstrated a significant reduction in the expression of M1 macrophage markers, such as iNOS and CD80, alongside an increase in M2 markers, including CD206 and Arg‐1. This change underscores the successful modulation of macrophage pathogenic phenotypes, indicating a regulatory effect on their functional state. In addition, an increasing body of evidence shows that metabolic pathways, specifically glycolysis, can lead to NLRP3 inflammasome activation.^[^ ^39^ ^]^ Vinaik et al. discovered a correlation between NLRP3 inflammasome activation and glucose transporter 1 (GLUT1)‐dependent glycolysis in postburn injuries.^[^ ^40^ ^]^ In parallel, Zhong et al. observed that inhibiting glycolysis partially reduced NLRP3 inflammasome activation in acute lung injury (ALI).^[^ ^41^ ^]^ In this context, we paid attention to the NLRP3 inflammasome and found that MLT‐MLP treatment did reduce the NLRP3 inflammasome activation, accompanied by the reduction of caspase 1 and IL‐1β release. However, the exact mechanism of how glycolysis activating the NLRP3 inflammasome remains ambiguous. One explanation is the involvement of hexokinase 1 (HK‐1) and hexokinase 2 (HK‐2), both of which are capable of interacting with the mitochondrial voltage‐dependent anion channel, thereby inducing the activation of NLRP3.^[^ ^42^ ^]^ Alternatively, the increase of ROS induced by glycolysis caused the activation of NLRP3 inflammasome.^[^ ^43^ ^]^ The exact mechanism by which glycolysis promotes NLRP3 inflammasome needs further study.

Then we conducted further research on the molecular regulatory mechanism of MLT‐MLP. mTOR serves as the principal regulator of cellular growth and metabolic state in response to nutrients, growth factors and a multitude of extracellular stimuli.^[^ ^44^ ^]^ A diverse array of extracellular stimuli, including growth factors, Toll‐like receptor (TLR) ligands and cytokines can activate mTOR pathway. Upon external stimuli, mTOR can be activated and impacts multiple aspects of glucose metabolism.^[^ ^45^ ^]^ For example, mTOR promotes glycolysis through induction and regulation of two critical transcription factors, hypoxia‐inducible factor 1α (HIF1a) and Myc. They are involved in different aspects of glycolysis including glucose transport and metabolism. For example, by modulating the expression of HIF1a and Myc, mTOR can increase the glucose transporter‐1 (GLUT1) expression to increase the uptake of glucose. mTOR also modulates the metabolic enzymes that control key steps in glycolysis, such as HK‐2, pyruvate kinase M2 (PKM2). Additionally, mTOR pathway influences various facets of mitochondrial metabolism, including oxygen consumption, membrane potential, and mitochondrial biogenesis.^[^ ^46^ ^]^ This change in the metabolism plays a critical role in the rapid response and energy supply of macrophages.^[^ ^45^ ^]^ So, we presumed that MLT‐MLP might regulate metabolism through mTOR pathway in macrophages. To test this hypothesis, we quantified the phosphorylation level of mTOR and its downstream effector, S6. S6 regulates cell growth and proliferation by selectively translating mRNAs of ribosomal proteins and enzymes involved in glycolysis and other metabolic pathways.^[^ ^47^ ^]^ We detected the levels of these key proteins in macrophages treated with MLT‐MLP and found that MLT‐MLP restored the metabolic balance in macrophages by reducing the phosphorylation of mTOR and S6.

In this study, we applied systemic administration approach to deliver MLT‐MLP instead of local delivery approach, such as intra‐articular injection and transdermal delivery. Gout is an acute inflammatory condition, and to achieve rapid suppression of inflammation, it is essential to accumulate a sufficient drug concentration at the site of inflammation in a short time. Intravenous injection allows the drug to directly enter the bloodstream and reach all tissues and organs rapidly, promoting rapid therapeutic effects. Additionally, as gout attacks also trigger systemic inflammation, serum inflammatory factors are also significantly increased during gout attacks. Therefore, intravenous administration can aid in alleviating this widespread inflammation. Although local administration has many advantages, such as reducing systemic side effects, its range of action is limited. The drug may have difficulty penetrating the skin or other physiological barriers when applied locally, leading to inadequate absorption or suboptimal effectiveness. In some cases, it can be challenging to precisely control the concentration and release rate of the drug in the localized area, which may impact the treatment outcome. Therefore, given the urgency and systemic nature of gout inflammation, intravenous administration might be the preferred method in our research.

Finally, the druggability and market feasibility of melatonin were also discussed here. First, melatonin exhibits strong anti‐inflammatory effects, making it a promising candidate for the treatment of various inflammation‐related conditions. Additionally, melatonin is generally considered safe for use, with a low incidence of side effects. This safety profile is crucial for long‐term treatment regimens and contributes to its attractiveness as a therapeutic agent. Moreover, its compatibility with various delivery systems (such as intravenous and transdermal) allows for versatile formulation options, which can enhance therapeutic efficacy and patient adherence. Additionally, with the increasing pace of life and rising prevalence of unhealthy lifestyle habits, the incidence of gouty arthritis is on the rise. There is a growing demand among patients for treatments that alleviate pain and improve quality of life. Melatonin, as a potential anti‐inflammatory agent, holds significant market potential. Its natural origin and perceived safety could further enhance its appeal to both patients and healthcare providers. Therefore, melatonin, as a potential anti‐inflammatory agent with favorable safety profile, holds significant market potential.

In conclusion, this study constructed a macrophage‐biomimetic melatonin‐loaded liposome (MLT‐MLP) for regulating the metabolic reprogramming of macrophages to treat acute gout. The MLT‐MLP ingeniously combined the natural inflammatory homing properties of macrophage membrane with the ability of melatonin to modulate macrophage metabolism. This not only achieved effective drug targeting to inflammatory sites but also exerted potent anti‐inflammatory effects. In acute gouty arthritis mice model, MLT‐MLP showed remarkable therapeutic effect, significant reducing the joint swelling. Mechanically, MLT‐MLP modulated the metabolic pattern of inflammatory macrophages by inhibiting the mTOR pathway, contributing to its therapeutic effect and providing a promising treatment strategy for gouty arthritis.

## 4 Experimental Section

### Ethical Regulations

All the animal experiments were performed in accordance with the guidelines evaluated and approved by the Ethics Committee of Shanghai Pudong Hospital (Ethical approval number: 2021‐DS‐Q‐26).

### Materials

Melatonin (MLT), colchicine (COL) were purchased from Aladdin Biochemical Technology Co., Ltd. (Shanghai, China). 1,2‐dimyristoyl‐sn‐ glycero‐3‐phosphocholine (DMPC) was purchased from AVT Pharmaceutical Tech Co., Ltd (Shanghai, China). 1,2‐distearoyl‐sn‐glycero‐3‐phosphoethanolamine‐N‐ [amino (polyethylene glycol)−2000] (ammonium salt) (DSPE‐PEG2000) was obtained from Xi'an Ruixi biotech (Xi'an, China). 1,1′‐dioctadecyl‐3,3,3′,3′‐ tetramethylindotricarbocyanine iodide (DiR), 1,1′‐dioctadecyl‐3,3,3′,3′‐tetramethylindocarbocyanine perchlorate (DiI) and Coomassie Brilliant Blue R250 were provided by Yeasen Biotechnology (Shanghai, China). Hoechst 333422, RIPA lysis buffer, protease and phosphatase inhibitor cocktail, 5 × SDS‐PAGE sample loading buffer and BCA protein assay kit were offered by Beyotime Biotechnology (Shanghai, China). Cell Counting Kit‐8 (CCK‐8), 2′,7′‐dichlorodihydrofluorescein diacetate (DCFH‐DA) and enhanced chemiluminescence (ECL) western blotting substrate were provided by Meilun Biotechnology (Dalian, China). Glucose Assay Kit, Lactic Acid Assay Kit, and ATP Determination Kit were purchased from Jiancheng Bioengineering Institute (Nanjing, China). Antibodies against Integrin α4 (A4054), Integrin β1 (A21234), MAC‐1 (A23508), GAPDH (A19056) were purchased from ABclonal (Shanghai, China). Antibodies against CD206 (ab64693), F4/80 (ab300421), iNOS (ab178945), TNF‐α (ab307164), IL‐6 (ab290735) and IL‐1β (ab283818) were provided by Abcam. Antibodies against phospho‐mTOR (5536), mTOR (2983), phospho‐S6 (4858) and S6 (5364), phospho‐ERK (9106), ERK (9102), phospho‐P38 (9211), P38 (8690), phospho‐P65 (3033), P65 (8242), phospho‐IKKα/β (2078), and IKKα (2682) were purchased from Cell Signaling Technology (Danvers, MA, USA). Antibodies against NLRP3 (AG‐20B‐0014B), caspase 1 (AG‐20B‐0042B) and ASC (AG‐25B‐0006PF) were purchased from AdipoGen Life Sciences (San Diego, CA, USA). Recombinant mouse TNF‐α were purchased from Novoprotein Biotechnology (Shanghai, China). FITC‐labeled anti‐CD80 antibody, PE‐labeled anti‐CD206 antibody, PE‐labeled anti‐CD54 (ICAM‐1) antibody and PE‐labeled anti‐CD106 (VCAM‐1) antibody were purchased from eBioscience (San Diego, CA, USA). Enzyme‐linked immunosorbent assay (ELISA) kits were provided by Multi Science (Hangzhou, China). Dulbecco's modified Eagle medium (DMEM), certified fetal bovine serum (FBS), penicillin‐streptomycin stock solutions, and trypsin‐EDTA (0.25%) were obtained from Invitrogen Co. (Carlsbad, CA, USA). Poly(dA:dT), FLA‐ST were offered by Invivogen (San Diego, USA). M‐CSF were provided with PeproTech (Cranbury, USA). Lipofectamine 3000‐Transfection Reagent (L3000015) was purchased from ThermoFisher (Wilmington, USA). The other chemical agents were purchased from Sigma‐Aldrich (St Louis, MO, USA).

### Cell Lines and Cell Culture

Immortalized bone marrow‐derived macrophages (iBMDMs) kindly provided by Prof. Kai Zhao (The Third Xiangya Hospital, Central South University, Changsha, Hunan), RAW264.7 cells and human umbilical vein endothelial cells (HUVECs), obtained from Chinese Academy of Science Cell Bank (Shanghai, China) were cultured with high glucose DMEM which contained 10% FBS (v/v), 100 U mL^−1^ penicillin−streptomycin at 37 °C with 5% CO2 in a humid atmosphere. Primary mouse bone marrow‐derived macrophages (BMDMs) were prepared and cultured as described previously.^[^ ^48^ ^]^ Briefly, bone marrow cells were extracted from the leg bones and differentiated in RPMI‐1640 (Gibco) with 10% (v/v) FBS containing M‐CSF (20 ng mL^−1^). The medium was substituted with fresh medium after 3 days. On day 6 or 7, BMDMs were harvested and replated for experiments.

### MSU Crystals Preparation

Uric acid (1 g) and sodium hydroxide (0.5 g) were dissolved in 100 mL of Milli‐Q water and subjected to heating at 80 °C for 20 min. Subsequently, the pH of solution was adjusted to 7.2 before being allowed to cool to room temperature. Following centrifugation (3 min at 1500 g), the precipitate was isolated. A portion of the aqueous phase was then evaporated, and the remaining samples were crystallized for 2 h at 180 °C to achieve sterilization of the sample. Ultimately, the crystals were preserved under aseptic conditions.

### Establishment of Acute Gouty Arthritis Model

Male C57B/6L mice, aged 6–8 weeks and weighing 20–25 g, were purchased from the BK Lab Animal Ltd. (Shanghai, China). For the establishment of the acute gout model, mice underwent administration of 50 µL of normal saline into the left paw and 1 mg of MSU crystals dissolved in 50 µL of normal saline into the right paw. Mice injected with normal saline in both left and right paws were designed as the control group.

### Preparation of MLT‐MLP

To isolate the macrophage membrane, RAW264.7 cells were collected and suspended in the ice‐cold TM buffer (pH 7.4; 0.01 M Tris + 0.001 M MgCl_2_) at a concentration of 2.0 × 10^7^ cells mL^−1^. After five freeze‐thaw cycles in liquid nitrogen, the cells were subjected to 30 passes in homogenizer and centrifuged at 10,000 g and 4 °C for 20 min to remove the precipitated intracellular components. The supernatant was then collected and further centrifuged at 10,0000 × g and 4 °C for 1 h. The final precipitate was collected as the purified macrophage membrane and stored at −80 °C for future study.^[^ ^49^ ^]^ Liposomes loaded with melatonin were prepared by thin film dispersion method. Briefly, DMPC, DSPE‐PEG2000, and melatonin were dissolved in chloroform at the mass of 25:5:3 and the chloroform was removed by a rotary evaporator under reduced pressure to obtain a uniform film, and then 5 mL PBS was added to hydrate at 37 °C for 30 min to obtain melatonin‐loaded liposomes (MLT‐LP). The membrane isolated from 1 × 10^8^ cells was mixed with 1 mL MLT‐LP, and then the mixture was extruded 20 times through the 400 nm and 200 nm polycarbonate porous membranes by an Avanti mini extruder to obtain macrophage membrane‐coated liposomes (MLT‐MLP). The DiI or DiR‐labeled nanoparticles were prepared with the same method by adding 1 ‰ DiI or DiR to the lipids.

### Characterization of MLT‐MLP

The size and zeta potentials of nanoparticles were determined by DLS using a Malvern ZetaSizer Nano series (Westborough, MA, USA), and the morphology were examined under transmission electron spectroscopy (TEM) (Tecnai G2 F20 S‐Twin) after negatively stained with phosphotungstic acid at 1% (w/v). The encapsulation efficiency (EE%) and loading capacity (LC%) of MLT were measured by HPLC. The mobile phase was composed of methanol and water at a ratio of 50:50 (v/v) and the detection wavelength was 222 nm. The membrane proteins of MLT‐MLP were identified by Coomassie blue staining and Western blot. Proteins in RAW264.7 cells, macrophage membrane, MLT‐LP, and MLT‐MLP were extracted with a RIPA lysis buffer and then mixed with SDS‐PAGE loading buffer and heated at 98 °C for 5 min. Then the lysates were centrifuged (10000 × g, 5 min, 4 °C) and the protein concentration of supernatants were quantified by enhanced BCA protein assay. For Coomassie blue staining, 30 µg protein of each sample was loaded into the SDS‐PAGE gel for electrophoresis followed by staining with Coomassie blue for 1 h and bleaching for 12 h before imaging. For Western blot, the proteins were transferred from SDS‐PAGE gel to nitrocellulose filter membrane after electrophoresis. The membranes were then blocked with 5% nonfat dry milk and incubated with anti‐Integrin α4 (1:1000), anti‐Integrin β1 (1:3000) and anti‐Mac‐1 (1:1000) at 4 °C overnight. The membranes were then incubated with HRP‐conjugated secondary antibody (1:10000) for 1 h at room temperature, and the blots were detected with ECL western blotting substrate and chemiluminescence imaging system. To investigate the in vitro MLT release profile, free MLT, MLT‐LP and MLT‐MLP were incubated in PBS with 0.5% (w/v) Tween 80 in a dialysis bag. 200 µL of solution was collected at different time points. Then the concentration of MLT was detected by HPLC. The condition of HPLC analysis was the same as described above.

### Cell Viability Assay

For evaluation of the viability of iBMDMs and HUVECs, the cell viability was measured by CCK8 kit according to the protocol at 24 h after administration of different formulations,.

### MLT‐MLP Uptake In Vitro

To test nanoparticles uptake by RAW264.7 cells, RAW264.7 cells were treated with DiI‐labeled MLT‐LP or DiI‐labeled MLT‐MLP for different hours and washed by PBS. Next, cells were fixed with 4% paraformaldehyde (PFA) for 15 min and the nuclei were stained with Hoechst 333422 for 10 min. Then, cells were observed under a fluorescence microscope (DMI4000D, Leica, Germany), and analyzed by ImageJ. To test nanoparticles uptake by inflammatory iBMDMs, DiI‐labeled MLT‐MLP were added to inflammatory iBMDMs and followed the above procedure.

### In Vitro Binding of MLT‐MLP

To evaluate the potential of macrophage membrane coating in enhancing the active targeting capability of MLT‐MLP, an in vitro binding assay was conducted. HUVECs were cultured on confocal dishes at a seeding density of 1 × 10^4^ per dish. Upon achieving 70−80% confluence, the culture was treated with TNF‐α (50 ng mL^−1^) for 6 h to simulate an inflamed vascular environment. Subsequently, DiI‐labeled MLT‐LP and DiI‐labeled MLT‐MLP were introduced to the cultures. To elucidate the binding mechanism, MLT‐MLP was pre‐incubated with 20 µL of anti‐integrin α4 antibody and 20 µL of anti‐Mac‐1 antibody per 1 mL of the preparation, to generate an antibody‐blocked variant of MLT‐MLP, termed blocked MLT‐MLP. Following a 4‐h incubation at 37 °C, the cells were subjected to triple washing with PBS. Nuclear staining was performed using Hoechst 333422, and the samples were examined with a fluorescence microscope. ImageJ software was utilized for the analysis of the results.

### In Vivo Targeting Assay

The mice with gout were divided into three groups, each receiving intravenous administration of DiR, DiR‐MLT‐LP, or DiR‐MLT‐MLP. Subsequent to the injection, the animals were anesthetized using isoflurane at predetermined intervals (2, 6, 12, 24, 48, and 72 h) for imaging via an in vivo imaging system (IVIS). Upon completion of the final imaging session, euthanasia was performed, and critical organs (heart, liver, spleen, lung, kidney, and paw) were harvested for further imaging. The near‐infrared fluorescence (NIRF) intensity within these tissues was quantitatively assessed through region‐of‐interest (ROI) analysis.

To explore the targeting mechanism, DiR‐labeled MLT‐MLP was administered to both gout‐afflicted and healthy mice. Two hours post‐injection, the right paw tissues were preserved in 4% paraformaldehyde, followed by a decalcification process. Tissue sections were then incubated with antibodies against ICAM‐1 or VCAM‐1 at 4 °C for 12 h. After dual washes, sections were treated with Alexa Fluor 488‐conjugated secondary antibodies for 30 min, then stained with DAPI. Fluorescence microscopy facilitated the examination of these samples, with ImageJ software being utilized for the analytical quantification.

### Establishment of Cellular Inflammation Model and Treatment

The initiation of the NLRP3 inflammasome requires a two‐step initiation process,^[^ ^50^ ^]^ so following a 3‐h pre‐treatment with 100 ng mL^−1^ LPS, a 6‐h stimulation with 200 µg mL^−1^ MSU was introduced to iBMDMs or BMDMs to establish an gouty inflammatory cell model. Different formulations (including PBS, MLT, MLT‐LP, and MLT‐MLP) were incubated with the cells for 12 h before establishing the model. The concentration of melatonin was 10 µM. Unless otherwise specified, macrophages were subjected to these conditions for induction of inflammation and treatment. The cells not subjected to inflammation induction were designated as the control group. To establish AIM2 inflammasome, BMDMs were transfected by poly (dA:dT) (1 µg mL^−1^, 16 h) by Lipofectamine 3000 following 3‐h priming with 100 ng mL^−1^ LPS. To establish NLRC4 inflammasome, BMDMs were transfected with flagellin (2 µg mL^−1^, 1 h) following 3‐h priming with 100 ng mL^−1^ LPS.

### Intracellular ROS Assay

DCFH‐DA served as the reagent for the intracellular ROS assay. Briefly, DCFH‐DA was introduced to the cellular milieu, followed by an incubation period in darkness at 37 °C for 30 min. Subsequently, the cells underwent washing with PBS to eliminate unincorporated DCFH‐DA probes. Thereafter, the samples were visualized using fluorescence microscopy and analyzed via flow cytometry to quantitatively assess the levels of intracellular ROS.

### Investigation of the Polarization Types of Macrophages

The iBMDMs were meticulously detached using a cell scraper while maintained on ice, followed by triple washing with pre‐cooled PBS. Subsequently, these cells were labeled with CD80 for 30 min at 4 °C. Post‐staining, cells underwent fixation and permeabilization employing a Fixation/Permeabilization Kit (Thermo Scientific), subsequent to be labeled with CD206 for an additional 30 min. To quantitatively evaluate the expression levels of CD80 and CD206, the cells were analyzed through flow cytometry.

### RNA‑seq

RNA‐seq was performed as described before.^[^ ^48a^ ^]^ Briefly, total RNA was obtained using an RNeasy mini kit (Qiagen). Strand‐specific libraries for RNA‐seq were prepared using the TruSeq stranded total RNA sample preparation kit (Illumina), quantified by Qubit 2.0 Fluorometer (Life Technologies), and validated for insert size by 2100 bio‐ analyzer (Agilent). Cluster was generated by cBot with the library diluted to 10 pM and subjected to sequencing using NovaSeq 6000 (Illumina). The library construction and sequencing were performed by Shanghai Biotechnology Corporation.

### Proteomic Analysis

Protein lysates were prepared for LC‐MS/MS analysis by Orbitrap Exploris 480 with a FAIMS coupled to an EASY‐nanoLC 1200 system (Thermo Fisher Scientific, MA, USA). Raw data were processed and analyzed by Spectronaut 14 (Biognosys AG, Switzerland) with default settings. The MS raw data were searched against the Uniprot fasta database within the default parameters. Qvalue (FDR) cutoffs on precursor and protein level were set at 0.01. Proteins significantly changed were included for GO and KEGG analysis.

### Immunofluorescence (IF) Staining

Briefly, the medium was removed and iBMDMs underwent triple washes with PBS, followed by fixation in 4% PFA for 10 min. Permeabilization was achieved with 0.1% Triton X‐100 for 15 min, and nonspecific binding sites were blocked using 3% bovine serum albumin (BSA) for 30 min. Subsequently, cells were incubated with F4/80 antibody, iNOS antibody, CD206 antibody or ASC antibody overnight at 4 °C following by staining Alexa Fluor 488 or Alexa Fluor 647‐labeled secondary antibody for 1 h at room temperature. Finally, nuclei were stained with Hoechst 333422 for 10 min at room temperature. Visualization of the cells was conducted using a confocal laser scanning microscope (CLSM).

### ELISA Assay

To detect the anti‐inflammatory effect of MLT‐MLP in vitro, inflammatory BMDMs were cultured with serum‐free medium containing different formulations. Following incubation, the culture supernatant was harvested, and the secretion levels of immune mediators from macrophages with different treatments were quantified utilizing ELISA kits according to the provided protocol. For the in vivo evaluation of the anti‐inflammatory potential of MLT‐MLP, serum samples from gout‐afflicted mice were collected 8 h subsequent to treatment administration. Thereafter, the concentrations of inflammatory cytokines in these samples were determined employing ELISA kits, following the specified protocol rigorously.

### Metabolite Assay

The glucose uptake was determined by the Glucose Assay Kit under manufacturer's direction. The lactate production was determined with Lactic Acid assay kit under manufacturer's direction. The intracellular ATP concentrations was determined by ATP Determination Kit under manufacturer's direction.^[^ ^48a^ ^]^

### Real‐Time Metabolism Analysis

The real‐time oxygen consumption rate (OCR) and extracellular acidification rate (ECAR) were quantified utilizing a Seahorse XF96 analyzer (Agilent Technologies, USA), according to the protocols provided by the manufacturer. Initially, iBMDMs were allocated into Seahorse 96‐well plates (Agilent Technologies, USA) for subsequent experimental conditions. For ECAR quantification, the medium comprised XF DMEM, enriched with 2 mm glutamine. Subsequently, a sequential injection of glucose (100 mM), oligomycin (10 µM), and 2‐deoxy‐D‐glucose (2‐DG, 500 mM) was administered at predefined intervals. Conversely, OCR assessment necessitated a preparatory environment of XF DMEM, supplemented with 10 mM glucose, 2 mM glutamine, and 1 mM pyruvate. Thereafter, a series of injections oligomycin (15 µM), carbonyl cyanide‐4 (trifluoromethoxy) phenylhydrazone (FCCP, 20 µM), and rotenone/antimycin A (5 µM) were dispensed into the microplate wells at designated junctures. Seahorse Wave software facilitated subsequent data interpretation.^[^ ^51^ ^]^

### Western Blot

Samples underwent extraction via RIPA lysis buffer, supplemented with phenylmethylsulfonyl fluoride and a phosphatase inhibitor cocktail. This process was conducted for 30 min, succeeded by centrifugation (10000 × g, 5 min, 4 °C). The supernatant was collected for total protein quantification using an enhanced BCA protein kit and heated with loading buffer at 98 °C for 5 min to prepare samples. Subsequently, the samples were transferred onto an SDS‐PAGE gel for electrophoretic separation, followed by transmembrane and blocking. The blots underwent sequential incubation with primary antibodies and horseradish peroxidase (HRP)‐conjugated anti‐rabbit IgG. Detection of all bands was achieved through the application of electrochemiluminescence western blot substrate, with quantitative analysis facilitated by ImageJ software. All the primary antibodies were diluted at 1:1000, unless otherwise specified.

### Dual Luciferase Reporter Gene Assay

RAW264.7 cells were transfected with a NF‐κB luciferase reporter plasmid and a pRL‐TK‐Renukka‐luciferase plasmid. 24 h after transfection, cells were subjected to the indicated treatment and then NF‐κB luciferase activities were measured using the Dual Luciferase Reporter Assay System (Promega), according to the manufacturer's instructions. Data are normalized for transfection effciency by dividing Firefly luciferase activity with the activity of Renilla luciferase.

### Assessment of In Vivo Biological Safety

After 7 days of various treatments on gout mice, 200 µL of whole blood was collected from the orbital sinus of each mouse. The blood was mixed with the anticoagulant EDTA and stored at 4 °C. Hematological parameters, including red blood cell count (RBC), platelet count (PLT), white blood cell count (WBC), percentage of neutrophils (Gran%), and percentage of lymphocytes (Lymph%), were analyzed using an automated hematology analyzer. To assess liver and kidney function, an additional 200 µL of blood was collected from the orbital sinus of each mouse without the use of anticoagulants. The blood samples were allowed to clot at room temperature for 2 h, after which they were centrifuged to obtain the serum. Liver function indicators, such as alanine aminotransferase (ALT) and aspartate aminotransferase (AST), as well as kidney function indicators, including UREA and serum creatinine (CREA), were measured according to the instructions provided with the respective assay kits. The mice were anesthetized with isoflurane and then euthanized. Major organs, including the heart, liver, spleen, lungs, and kidneys, were harvested. These organs were then embedded in paraffin, sectioned into thin slices, and stained with H&E. Finally, pathological changes in the major organs were observed under an inverted fluorescence microscope.

### Statistical Analysis

All data are presented as the mean ± standard deviation (SD) and were subjected to analysis utilizing Prism software version 9.0.0. Statistical evaluations were conducted employing one‐way Analysis of Variance (ANOVA) for the comparison across multiple groups. Levels of statistical significance were denoted as ^*^ *p* < 0.05, ^**^ *p* < 0.01, and ^***^ *p* < 0.001, as depicted in the Figure captions.

## Conflict of Interest

The authors declare no conflict of interest.

## Supporting information



Supporting Information

## Data Availability

The data that support the findings of this study are available from the corresponding author upon reasonable request.
