# Supplementary material for: Metabolic Reprogramming of Macrophages by Biomimetic Melatonin‐Loaded Liposomes Effectively Attenuates Acute Gouty Arthritis in a Mouse Model
Source: Adv Sci (Weinh). 2024 Dec 24;12(7):2410107. doi: 10.1002/advs.202410107 (PMC11831490; doi:10.1002/advs.202410107)

Supporting Information

**Metabolic reprogramming of macrophages by biomimetic melatonin-loaded liposomes effectively attenuates acute gouty arthritis in a mouse model**

*Chuchu Ma, Yuyu Jiang, Yan Xiang, Chang Li, Xiaoying Xie, Yunkai Zhang, Yang You, Laozhi Xie, Jianing Gong,* *Yinzhe Sun, Shiqiang Tong, Qingxiang Song, Jun Chen**^*^, Wenze Xiao^*^*

C. Ma, C. Li, X. Xie, Y. You, L. Xie, J. Gong, Y. Sun, S. Tong, J. Chen

Department of Pharmaceutics, School of Pharmacy & Shanghai Pudong Hospital, Key Laboratory of Smart Drug Delivery, Ministry of Education, Fudan University, Shanghai 201203, China

E-mail: chenjun@fudan.edu.cn

W. Xiao

Department of Rheumatology, Shanghai Pudong Hospital, Fudan University Pudong Medical Center, Shanghai 201399, China

E-mail: wenzexiao@fudan.edu.cn

Y. Zhang

Naval Medical Center, Naval Medical University, Shanghai 200433, China

Y. Jiang, Y. Xiang

Department of Pathogen Biology, Naval Medical University, Shanghai 200433, China

Q. Song

Department of Pharmacology and Chemical Biology, State Key Laboratory of Oncogenes and Related Genes, Shanghai Universities Collaborative Innovation Center for Translational Medicine, Shanghai Jiao Tong University School of Medicine, Shanghai 200025, China

**Supplementary Figures**

**
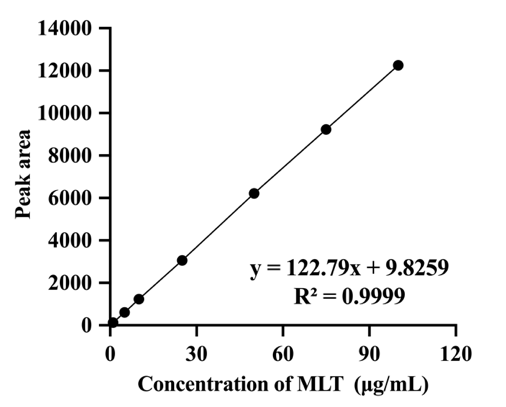
**

**Figure S1.** Standard curve of the concentration of MLT vs. peak area in HPLC


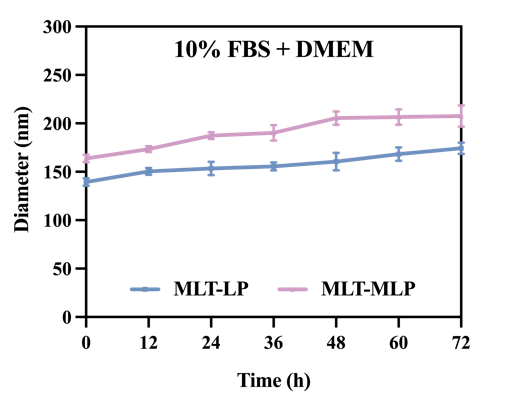


**Figure S2.** The stability of MLT-LP and MLT-MLP in DMEM with 10 % FBS (*n* = 3). Data are presented as mean ± SD.

**
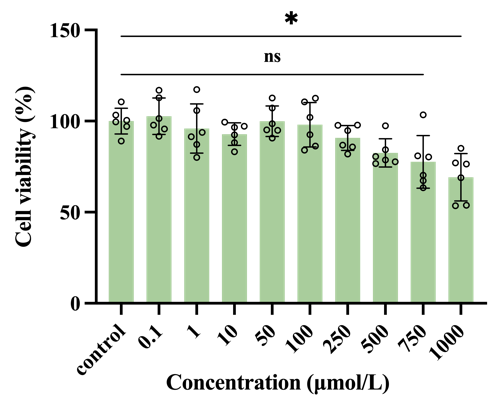
**

**Figure S3.** Assessing cytotoxicity in iBMDMs. Cytotoxicity was assessed at different concentration of MLT (*n* = 6). Data are presented as mean ± SD. **p* < 0.05; ns, not significant.


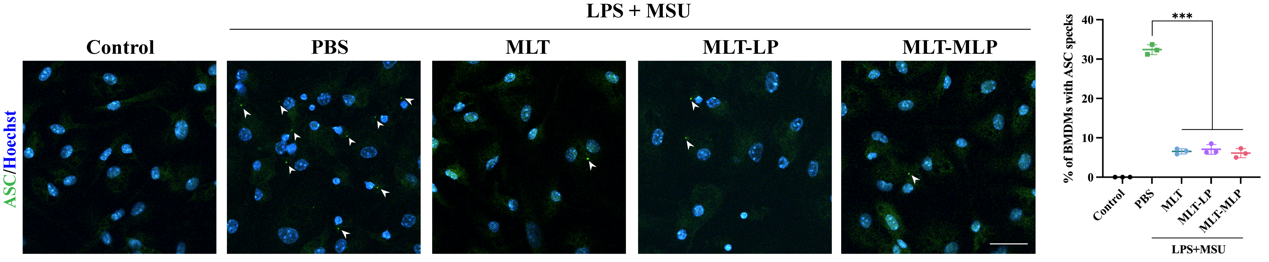


**Figure S4.** Immunofluorescence analysis and the statistics analysis of ASC specks formation in BMDMs stimulated with LPS+MSU or not, with different treatments. White arrows: ASC specks. Scale bar, 25 μm. Data were presented as mean ± SD (*n =* 3). ****p* < 0.001.


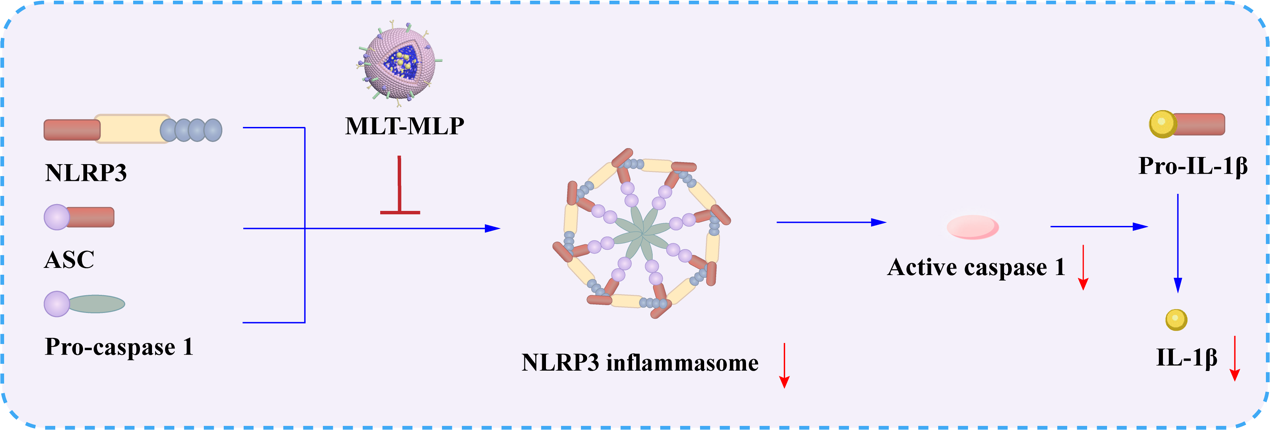


**Figure S5.** Illustrates the mechanisms underlying the control of NLRP3 inflammasome activation by MLT-MLP treatment.


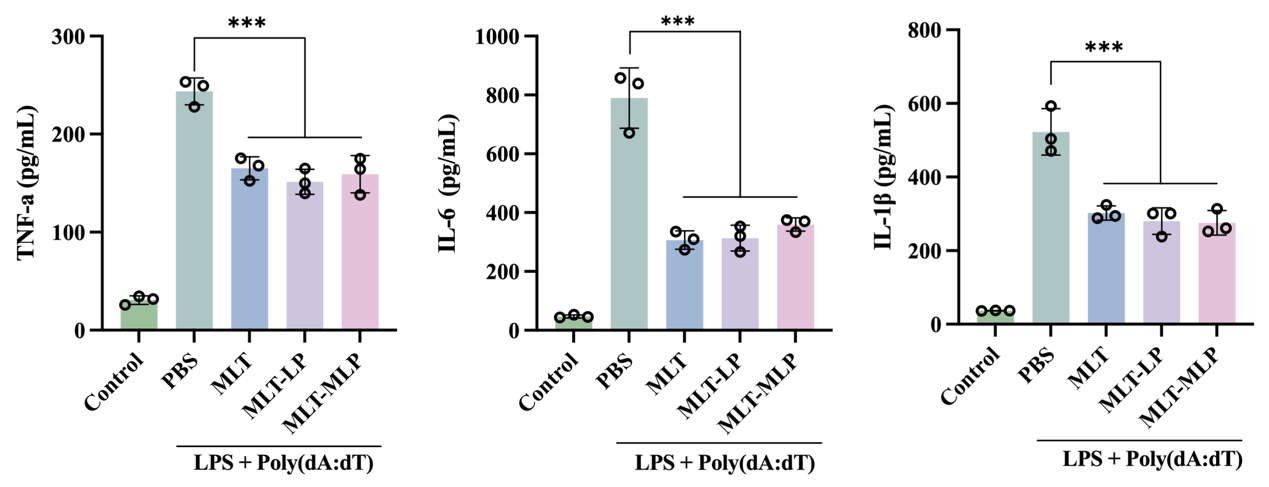


**Figure S6.** Detection of inflammatory cytokines (TNF-α, IL-6 and IL-1β) in the supernatant of BMDMs with different treatments (*n* = 3). To induce AIM2 inflammasome, BMDMs were first primed with 100 ng/mL LPS for 3 hours, followed by transfection with poly(dA:dT) (1 μg/mL) for 16 hours using Lipofectamine 3000. Data are presented as mean ± SD. ****p* < 0.001.


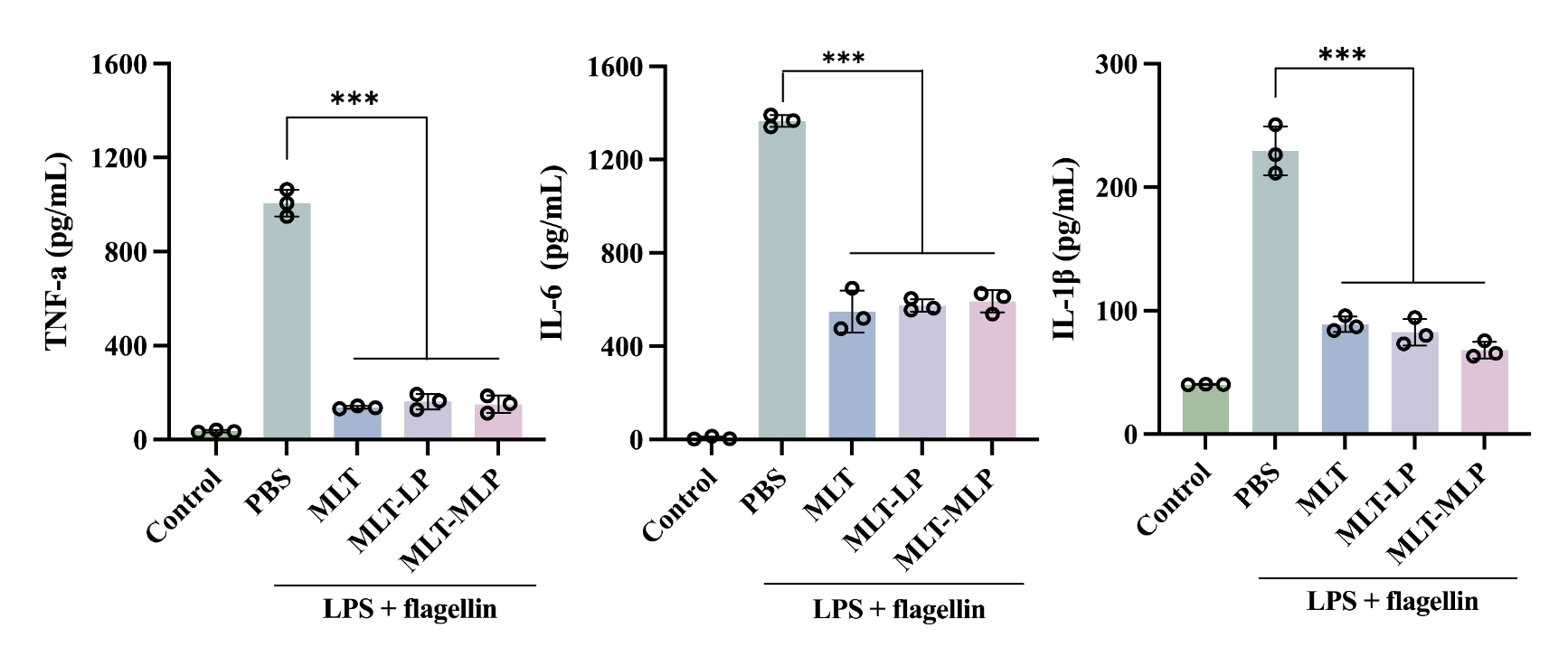


**Figure S7.** Detection of inflammatory cytokines (TNF-α, IL-6 and IL-1β) in the supernatant of BMDMs with different treatments (*n* = 3). To activate the NLRC4 inflammasome, BMDMs were initially primed with 100 ng/mL LPS for 3 hours, followed by transfection with flagellin (2 μg/mL) for 1 hour. Data are presented as mean ± SD. ****p* < 0.001.


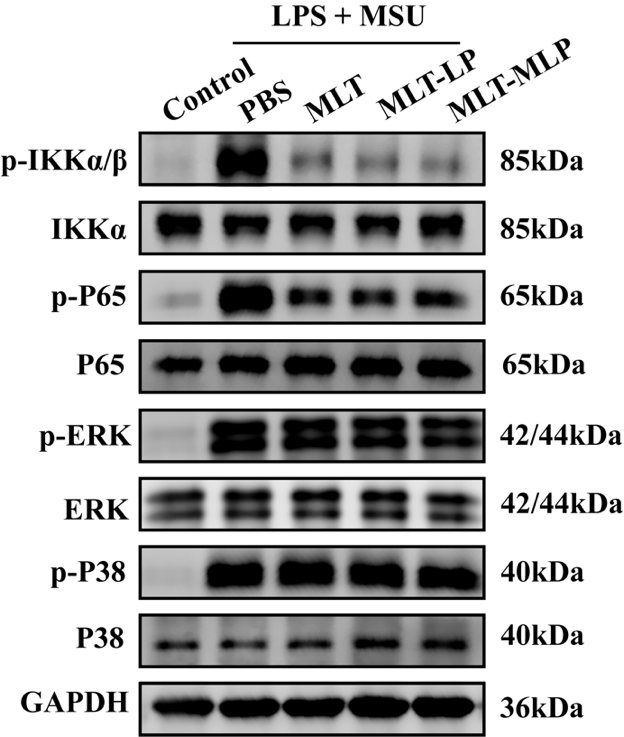


**Figure S8.** Western blot analysis of NF-κB and MAPK signaling pathway in BMDMs stimulated with LPS+MSU or not, with different treatments.


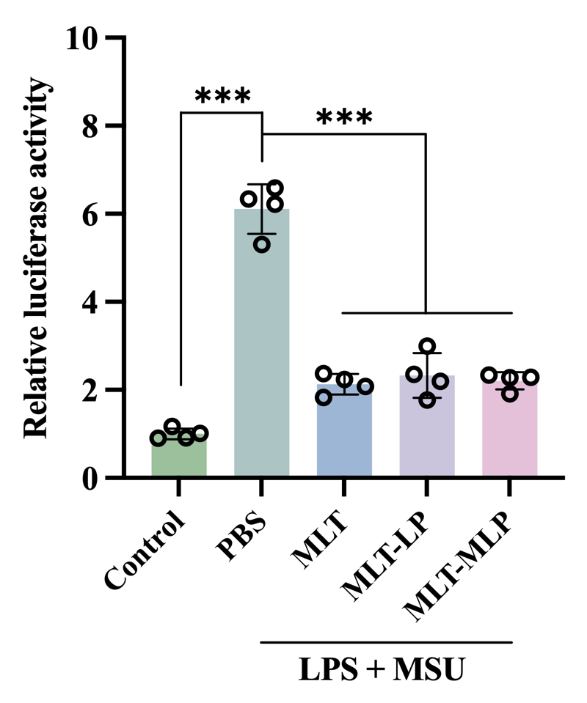


**Figure S9.** Dual luciferase reporter gene analysis of NF-κB-luciferase activity in RAW264.7 cells stimulated with LPS+MSU or not, with different treatments 24 hours after transfection with NF-κB-luciferase reporter plasmid (NF-κB-luci) and pRL-TK-Renukka-luciferase plasmid. Data were presented as mean ± SD (*n* = 4). ****p* < 0.001.


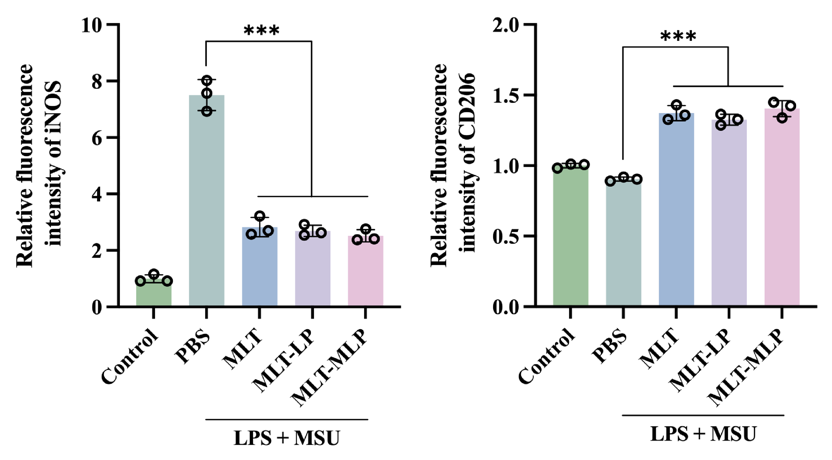


**Figure S10.** Fluorescence intensity quantitation of iNOS and CD206 in iBMDMs with different treatments corresponding to Figure 4D (*n* = 3). Data were presented as mean ± SD. ****p* < 0.001.


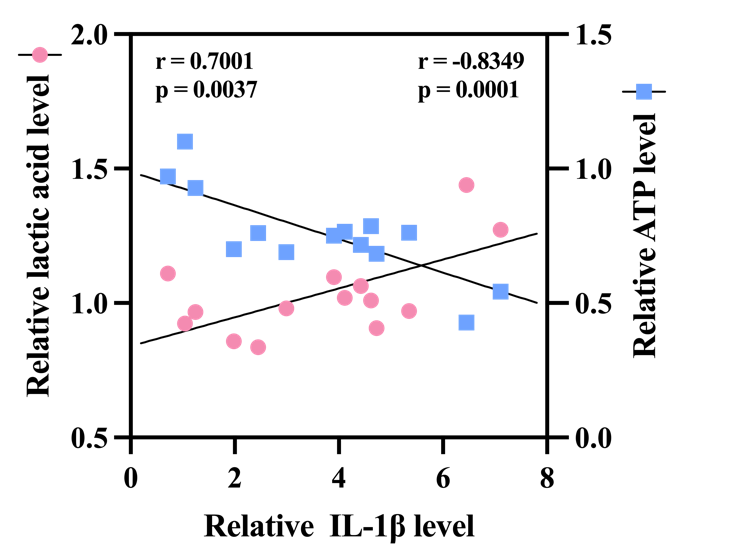


**Figure S11.** Correlation analysis of relative lactic acid and ATP levels versus IL-1β levels.

**Supplementary Table**

**Table S1.** Characterization of MLT-LP and MLT-MLP. Data were presented as mean ± SD (*n* = 3).


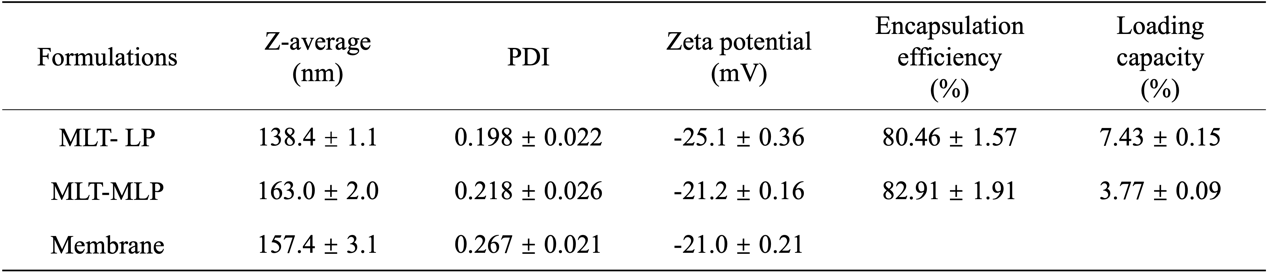

Supplement: Supplementary file 1 — Supporting Information [file ADVS-12-2410107-s001.docx]
